# Expression of and correlational patterns among neuroinflammatory, neuropeptide, and neuroendocrine molecules from cerebrospinal fluid in cerebral palsy

**DOI:** 10.1186/s12883-021-02333-2

**Published:** 2021-10-04

**Authors:** Cory J. Goracke-Postle, Chantel C. Burkitt, Angela Panoskaltsis-Mortari, Michael Ehrhardt, George L. Wilcox, Patrick Graupman, Michael Partington, Frank J. Symons

**Affiliations:** 1 College of Pharmacy, University of Minnesota, Minneapolis, MN 55455 USA; 2 Gillette Children’s Specialty Healthcare, Saint Paul, MN 55101 USA; 3 Department of Pediatrics, University of Minnesota Medical School, Minneapolis, MN 55455 USA; 4 Departments of Neuroscience, Pharmacology, Dermatology, University of Minnesota Medical School, Minneapolis, MN 55455 USA; 5 Children’s Mercy, Kansas City, MO 64108 USA; 6 Department of Educational Psychology, College of Education and Human Development, Minneapolis, MN 55455 USA

**Keywords:** Cerebral palsy, Neuroinflammation, Neuroendocrine, Nociceptive, Biomarkers, CSF, Children

## Abstract

**Background:**

The underlying pathogenesis of cerebral palsy (CP) remains poorly understood. The possibility of an early inflammatory response after acute insult is of increasing interest. Patterns of inflammatory and related biomarkers are emerging as potential early diagnostic markers for understanding the etiologic diversity of CP. Their presence has been investigated in plasma and umbilical cord blood but not in cerebrospinal fluid (CSF).

**Methods:**

A clinical CP sample was recruited using a single-time point cross-sectional design to collect CSF at point-of-care during a standard-of-care surgical procedure (intrathecal pump implant). Patient demographic and clinical characteristics were sourced from medical chart audit.

**Results:**

Significant (*p* ≤ 0.001) associations were found among neuroinflammatory, neuroendocrine, and nociceptive analytes with association patterns varying by birth status (term, preterm, extremely preterm). When between birth-group correlations were compared directly, there was a significant difference between preterm and extremely preterm birth subgroups for the correlation between tumour necrosis factor alpha (TNFα) and substance P.

**Conclusion:**

This investigation shows that CSF can be used to study proteins in CP patients. Differences in inter-correlational patterns among analytes varying by birth status underscores the importance of considering birth status in relation to possible mechanistic differences as indicated by biomarker signatures. Future work should be oriented toward prognostic and predictive validity to continue to parse the heterogeneity of CP’s presentation, pathophysiology, and response to treatment.

**Supplementary Information:**

The online version contains supplementary material available at 10.1186/s12883-021-02333-2.

## Background

Cerebral palsy (CP) is a neurodevelopmental disorder of movement, muscle tone or posture caused by an insult to the developing brain before birth, at birth, or before the age of two years. CP is the most common motor disability in childhood, affecting an estimated 500,000 American children and occurring in approximately 2 children per 1,000 live births. The pathogenesis of CP is not fully understood. CP risk factors predominantly involve perinatal factors such as anoxia and ischemia and prenatal factors such as young gestational age, intrauterine viral infections, and maternal thyroid abnormalities. Intrauterine infection and inflammation are of particular interest with both maternal response (chorioamnionitis) and fetal response (funicitis or elevated interleukin-6 in fetal plasma) being associated with white matter damage (WMD) and CP [1]. Injury and related inflammatory processes may persist for considerable periods of time (years) leading to hypotheses and emerging models of tertiary-like mechanism of damage including epigenetic regulation and inflammatory relevant changes [2].

Work investigating infection- and neuroinflammatory-related biomarkers related to WMD in neonates has generated evidence mostly supporting a pathway from intrauterine infection to placental inflammation then to systemic fetal circulation and the preterm newborn brain [3]. The specifics of the infection-inflammation-brain damage link have been described and documented and are an active area of investigation [4] particularly given cautions about what level of inference is or is not supported by high quality evidence [5]. What is less clear, however, is the robustness of the inference supporting an infection-inflammation-WMD link with CP as an inevitable outcome. There is some evidence for such a link [6], but it may be conditioned on whether the infant was born term or preterm. From a more general perspective, the emerging viewpoint on the role of neuro-inflammation as a pathophysiological contributor to CP has created the possibility of a new therapeutic window through which to view the condition [7, 8].

Specific findings from prior clinical work specifically investigating inflammatory mediators have demonstrated differential biomarker patterns in umbilical cord serum from infants stratified by preterm and CP status [1]. Of the potential inflammation markers that differed between cases and controls, the markers were lower (based on medians) in the preterm CP cases relative to controls but were higher, relative to controls, in the term CP cases. Sampling from a different compartment (plasma) and using a different approach, a study by Lin et al. (2010) comparing school-age children who were former preterm births reported higher cytokine responses (increased tumor necrosis factor-alpha [TNF-α] plasma levels and greater mRNA levels of toll-like receptor four [TLR4]) among those children with CP who were preterm relative to controls (children born preterm with normal development) [9]. Another study of blood, using a serial approach (i.e., repeated measures) in over 900 preterm infants, documented elevated concentration values of myriad inflammatory-relevant mediators which were associated with different risk profiles depending on the sampling day [10]. Using CSF from preterm infants with brain injury, Douglas-Escobar and Weiss (2012) documented combinations of biomarker concentration values that could be used to inform clinical decision making [4]. Using blood from a sample of children with and without CP, Zareen (2020) [11] found significantly increased levels of erythropoietin at baseline in children with CP compared with children in the comparison group. In response to challenge (lipopolysaccharide), both groups had appropriate and comparable response profiles for interleukin-8, vascular endothelial growth factor (VEGF), TNF-α, and granulocyte–macrophage colony-stimulating factor (GM-CSF) levels. The children with CP showed a statistically significant lipopolysaccharide hypo-responsiveness profile for interleukin-1a, interleukin-1b, interleukin-2, and interleukin-6 levels. Collectively, the work to date consistently shows immune and inflammatory differences in children with CP.

There remain gaps in our knowledge about the specific linkages among various immune and related mechanisms driving hypothesized persistent inflammatory states in children with CP, and the relation among the various biomarkers, risk factors, and specific outcomes. The overall generality of the findings to date for the CP population is limited by two kinds of problems, namely the relative difficulties in establishing valid pre-clinical models for this purpose [12] and the extreme paucity of clinically-relevant biomarker research within this high-need vulnerable patient group. For example, the relation among the CSF biomarkers investigated by Douglas-Escobar and Weiss (2012) and CP as an outcome was not clear [4]. More biomarker data of a comparable kind from the same and different compartments across the same and different age groups are needed. To the best of our knowledge, no comparable work has investigated inflammatory-relevant molecular biomarkers in CSF from children, adolescents, and young adults with CP.

The purpose of this preliminary investigation was exploratory. The design was cross-sectional using a single time point for specimen collection from a clinical sample. There were two specific aims. The first aim was to document levels of inflammatory and related molecules in CSF in a sample primarily of school-age children with CP. To do so, for detectable analytes, participants were arrayed along each analyte’s concentration gradient. The second aim was to examine clinically relevant grouping variables (e.g., CP severity, term/preterm birth) to identify any potentially relevant correlational patterns among the molecules. Our intent was to extend the work initiated by Kaukola and Lin (described above) using a clinical sample in which standard-of-care surgical interventions were leveraged to gain access to CSF for future hypothesis-generating research purposes.

## Methods

### Protocol approval

This study was approved by the Institutional Review Board (IRB, #0809M46301) of the University of Minnesota. Written informed consent was obtained from each participant or legal representative (i.e., parent/guardian).

### Participants

This study utilized a single-time point cross-sectional design. Twenty-eight individuals (82% male) with CP participated (mean age = 9.74 years, SD = 4.36; range = 4–23). Specific CP diagnoses included: quadriplegia (*n* = 17), diplegia (*n* = 5), and triplegia (*n* = 5). Participants were included in the study if they (a) had cerebral palsy, (b) were between 3–25 years of age, and (c) were scheduled for initial intrathecal baclofen (ITB) pump implant. Individuals were excluded if (a) they had an existing cerebral shunt; or (b) they had compounded dosing (i.e., opioid adjunctive to baclofen) through their pump. The participants were already characterized clinically using the Gross Motor Functional Classification System for Cerebral Palsy (GMFCS) to categorize gross motor function. The GMFCS is a 5-level classification system based on self-initiated movement with emphasis on truncal control and walking. The GMFCS is widely used clinically and in classifying individuals with CP for research studies [13].

For the subgroup comparisons, the breakdown of participant demographics was as follows: males (*n* = 23) and females (*n* = 5); non-quadriplegia (*n* = 11) and quadriplegia (*n* = 17); Caucasian (*n* = 26) and other (*n* = 2); spastic CP (*n* = 13) and mixed tone CP (*n* = 12); term birth, defined as 37 weeks or later (*n* = 6), preterm birth, defined as 28–37 weeks (*n* = 12) and extremely preterm birth, defined as less than 28 weeks (*n* = 9); seizure (*n* = 6) and no seizure (*n* = 16) (Table 1).

**Table 1:** Participant health information; M ± SD or n (%)

	Complete sample (*n* = 28)	Term birth (*n* = 6)	Preterm birth (*n* = 12)	Extremely preterm birth (*n* = 9)
Male	23 (82.1)	3 (50.0)	11 (91.7)	9 (100)
Ethnicity				
Caucasian	26 (92.9)	6 (100.0)	12 (100.0)	8 (88.9)
African American	1 (3.6)	0 (0)	0 (0)	1 (11.1)
Other (not specified)	1 (3.6)	0 (0)	0 (0)	0 (0)
Epilepsy				
None	16 (57.1)	3 (50.0)	7 (58.3)	6 (66.7)
History of seizure/diagnosis of epilepsy	6 (21.4)	1 (16.7)	4 (33.3)	1 (11.1)
Questionable seizure activity	3 (10.7)	1 (16.7)	0 (0)	1 (11.1)
Missing	3 (10.7)	1 (16.7)	1 (8.3)	1 (11.1)
CP topography				
Hemiplegia	1 (3.6)	0 (0)	1 (8.3)	0 (0)
Diplegia	5 (17.9)	2 (33.3)	3 (25.0)	0 (0)
Triplegia	5 (17.9)	1 (16.7)	0 (0)	4 (44.4)
Quadriplegia	17 (60.7)	3 (50.0)	8 (66.7)	5 (55.6)
GMFCS				
Level I	3 (10.7)	0 (0)	3 (25.0)	0 (0)
Level II	3 (10.7)	1 (16.7)	0 (0)	2 (22.2)
Level III	7 (25.0)	3 (50.0)	2 (16.7)	2 (22.2)
Level IV	8 (28.6)	1 (16.7)	4 (33.3)	3 (33.3)
Level V	6 (21.4)	1 (16.7)	2 (16.7)	2 (22.2)
Missing	1 (3.6)	0 (0)	1 (8.3)	
Tone				
Spastic	13 (46.4)	4 (66.7)	5 (41.7)	4 (44.4)
Mixed tone	12 (42.9)	2 (33.3)	5 (41.7)	4 (44.4)
Missing	3 (10.7)	0 (0)	2 (16.7)	1 (11.1)
Current feeding tube				
Yes	8 (28.6)	2 (33.3)	2 (16.7)	4 (44.4)
No	19 (67.9)	4 (66.7)	10 (83.3)	5 (55.5)
Missing	1 (3.6)	0(0)	0 (0)	0 (0)
Days in NICU at birth Range of NICU days	76.25 ± 67.04 (0–300)	3.50 ± 7.00 (0–14)	52.73 ± 29.72 (10–120)	137.33 ± 64.76 (90–300)

*Note*: gestational age was not available for one participant; Term birth = born 37 weeks gestation or later; preterm birth = born at 28–37 weeks gestation; extremely preterm birth = born at less than 28 weeks gestation; *CP *Cerebral Palsy, *GMFCS *Gross Motor Function Classification System, *level I *ambulant without assistance, *level II* ambulant without assistive devices, limitations outside the home, *level III* ambulant with assistive devices, wheelchair required outside the home, *level IV* non-ambulatory, self-mobile in wheelchair with limitations, *level V* non-ambulatory, self-mobility very limited

### CSF collection

Patients were consented in accordance with an approved IRB protocol. If consent was given, CSF was collected during a standard-of-care surgical procedure (ITB pump implant). In all cases, the surgery proceeded as usual until the spinal catheter had been placed. Then, the neurosurgeon collected 10–20 ml of CSF from the spinal catheter placed well above the spinal puncture site. This method avoided contaminating the collected CSF with blood.

Immediately following collection, the CSF was placed on wet ice (+ 4 °C) and transported to a cold room for processing, centrifuged at 3000 rpm × 5 min, pipetted into 100 and 250 µL aliquots, flash frozen in liquid nitrogen and archived at -80 °C. After specimen collection, the patient was monitored closely following routine operative and post-operative procedures. There were no adverse events.

### CSF analyte analysis

CSF was analyzed using conventional biochemical methods based on commercially available enzyme-linked immunosorbent assay (ELISA) kits and expression levels of each marker were quantified. Specifically, samples were tested by the Cytokine Reference Laboratory (CRL, University of Minnesota). This is a CLIA’88-licensed facility (license #24D0931212). Samples were analyzed for adrenocorticotropic hormone (ACTH), agouti-related peptide (AgRP), brain-derived neurotrophic factor (BDNF), ciliary neurotrophic factor (CNTF), follicle-stimulating hormone (FSH), growth hormone (GH), luteinizing hormone (LH), prolactin (PRL), and thyroid stimulating hormone (TSH) using the “Human Brain-Derived Protein Panel” on the Luminex platform and done as a multi-plex (Luminex instrument—Bioplex 100 [Bio-Rad, 1000 Alfred Nobel Drive, Hercules, CA, 94547], Software: bio-plex Manager 4.0). The polystyrene bead set (cat. # HPT-66 K-09) with kit lot number 1757143 was used. Kits/reagents were purchased from EMD Millipore Corporation, Billerica, MA. Interferon α2 (IFNα2), interleukin-1α (IL-1α), interleukin-1ra (IL-1ra), interleukin-6 (IL-6), interleukin-8 (IL-8), interleukin-10 (IL-10), interleukin-12p40 (IL-12p40), interleukin-12p70 (IL-12p70), interferon gamma-induced protein 10 (IP-10), monocyte chemotactic protein (MCP-1), macrophage inflammatory protein 1β (MIP1β), regulated on activation normal T expressed and secreted (RANTES), and tumor necrosis factorα (TNFα) were analyzed using the “Cytokine/Chemokine Panel 1” on the Luminex platform and done as a multi-plex (Luminex instrument—Bioplex 100 [Bio-Rad, 1000 Alfred Nobel Drive, Hercules, CA, 94547], Software: bio-plex Manager 4.0). The polystyrene bead set (cat. # MPXHCYTO-60 K-14) with kit lot number 1757142 was used. Kits/reagents were purchased from EMD Millipore Corporation, Billerica, MA. Dynorphin A, neuropeptide Y, somatostatin, β endorphin, cortisol, neurotensin, orexin A, substance P, melatonin, oxytocin, and melanocyte-stimulating hormone (α-MSH) were analyzed using the “Human Neuropeptide Panel” on the Luminex platform and done as a multi-plex (Luminex instrument—Bioplex 100 Bio-Rad, 1000 Alfred Nobel Drive, Hercules, CA, 94547], Software: bio-plex Manager 4.0). The polystyrene bead set (cat. # HNP-35 K-08) with kit lot number 1823005 was used. Kits/reagents were purchased from EMD Millipore Corporation, Billerica, MA.

Samples were assayed according to manufacturer’s instructions. ELISA employ the quantitative sandwich enzyme immunoassay technique. The absorbance is measured on the microtiter plate reader (Bio-Rad model 550). The intensity of the color formed is proportional to the concentration of the sample. Fluorescent color-coded beads coated with a specific capture antibody were added to each sample. After incubation, and washing, biotinylated detection antibody was added, followed by phycoerythrin-conjugated streptavidin. The beads were read on a Luminex instrument (Bioplex 100) which is a dual-laser fluidics-based instrument. One laser determines the analyte being detected via the color coding; the other measures the magnitude of the PE signal from the detection antibody which is proportional to the amount of analyte bound to the bead. Samples were tested in duplicate and values were interpolated from 5 parameter-fitted (5PL) standard curves.

### Statistical analyses

Data analysis was exploratory and relied on visual analysis, descriptive statistics, and correlational analyses. First, visual analysis of each analyte was conducted to understand its distributional form and to identify potential outliers. Further, measures of central tendency (means, medians) and variation (standard deviations, coefficients of variation) were calculated for each analyte.

Second, to understand the associations between analytes a series of pairwise scatterplots and Pearson product-moment correlations were computed between each possible pair of analytes for the entire sample. The correlations were tested for statistical significance against the null hypothesis of *r* = 0. Parallel analyses and plots were generated for the data set with missing data imputed with the lower limit / sensitivity number. Given the large number of correlations tested (528), Type 1 errors were controlled for by using the false discovery rate correction discussed by Benjamini & Hochberg (1995). Correlations were considered against an alpha = 0.05, after the false discovery rate correction was applied.

Third, to understand how the associations between analytes varied by subgroups, the correlational analyses described above were repeated for each of the gestational term subgroups. Given the large number of comparisons involved in these analyses, an alpha = 0.001 was set for each test after the false discovery correction rate was applied.

Finally, differences in correlations *between* subgroups were also tested. To be included in between-group analysis, each correlation first had to be statistically significant within the subgroups. Thirty-two pairs of correlations were statistically significant across all subgroups. Then the identified correlations were tested against one another to check whether they were significantly different by birth status. To do this, Fisher's r to z transformation was used to calculate the difference in correlations that met the criteria for inclusion, and tested for statistical significance of that difference; p ≤ 0.05.

## Results

### CSF analyte expression in CP subjects

**Fig. 1:**
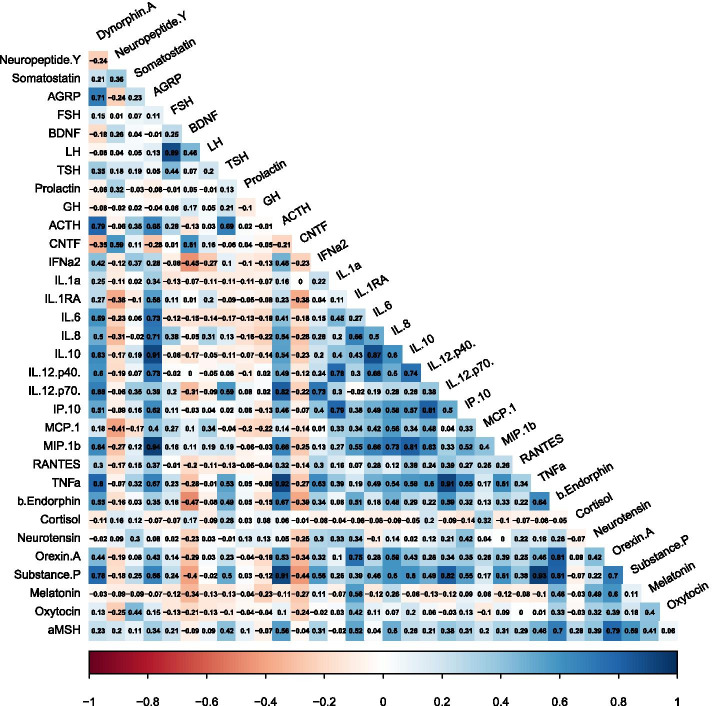
Visual representation of the direction and strength of the Pearson’s correlation coefficients between all 33 analytes assayed. Positive (blue), negative (red), strong (dark shading), and weak (light shading) correlations are depicted

Detectable CSF analytes were broken down, broadly, by the following categories: *hormonal/endocrine brain-derived peptides or proteins*: ACTH, AgRP, BDNF, CNTF, FSH, GH, LH, PRL, and TSH; *inflammatory cytokines/chemokines*: IL-1α, IL-1ra, IL-6, IL-8, IL-10, IL-12p40, IL-12p70, TNFα, IFN-α2, IP-10, MCP-1, MIP1β, and RANTES; and *neurotransmitters/neuropeptides*: Dynorphin A, neuropeptide Y, somatostatin, β endorphin, cortisol, neurotensin, orexin A, substance P, melatonin, oxytocin, melanocyte-stimulating hormone (α-MSH). Figure 1 illustrates the correlations among analytes (at *p* ≤ 0.001); a full list of analytes that demonstrated correlations (*p* ≤ 0.05) among the participants with CP is included in the Supplemental Information (Supplemental Table 1).

Considering this initial complete cohort, there were 35 analyte pairings with positive correlations as per our criteria from 21 distinct analytes (all *p* ≤ 0.001; Table 2). These 35 correlations represent combinations of endocrine, inflammatory, and excitatory neuropeptides. The specific correlation pattern represents a novel approach to considering analytes that may shed light on the mechanistic underpinnings of secondary processes that may be ongoing in CP and result in clinical signs and their manifestation.

**Table 2:** Significant Pearson’s correlations (*p* ≤ 0.001) between analyte pairs, representing 21 distinct analyte correlations (bold font)

Analyte 1	Analyte 2	Correlation	95% CI	Adj. p
**Dynorphin A**	**AGRP**	0.71	[0.34, 0.85]	< .001
Dynorphin A	**ACTH**	0.79	[0.60, 0.90]	< .001
Dynorphin A	**IL-12p70**	0.68	[0.41, 0.84]	0.001
Dynorphin A	**TNFα**	0.80	[0.60, 0.90]	< .001
Dynorphin A	**substance P**	0.78	[0.57, 0.89]	< .001
AGRP	**IL-6**	0.73	[0.49, 0.87]	< .001
AGRP	**IL-8**	0.71	[0.46, 0.86]	< .001
AGRP	**IL-10**	0.91	[0.81, 0.96]	< .001
AGRP	**IL-12p40**	0.73	[0.50, 0.87]	< .001
AGRP	**MIP-1β**	0.94	[0.87, 0.97]	< .001
AGRP	TNFα	0.67	[0.40, 0.84]	0.001
**FSH**	**LH**	0.89	[0.78, 0.95]	< .001
**TSH**	ACTH	0.69	[0.42, 0.84]	0.001
ACTH	IL-12p70	0.82	[0.65, 0.92]	< .001
ACTH	TNFα	0.92	[0.83, 0.96]	< .001
ACTH	**β endorphin**	0.67	[0.40, 0.83]	0.001
ACTH	substance P	0.91	[0.81, 0.96]	< .001
**IFNα2**	IL-12p70	0.73	[0.49, 0.87]	< .001
**IL-1α**	IL-12p40	0.78	[0.57, 0.89]	< .001
IL-1α	**IP-10**	0.79	[0.60, 0.90]	< .001
**IL-1RA**	**orexin A**	0.75	[0.52, 0.88]	< .001
IL-6	IL-10	0.87	[0.74, 0.94]	< .001
IL-6	IL-12p40	0.68	[0.41, 0.84]	0.001
IL-8	MIP-1β	0.73	[0.49, 0.87]	< .001
IL-10	IL-12p40	0.74	[0.51, 0.87]	< .001
IL-10	MIP-1β	0.81	[0.63, 0.91]	< .001
IL-12p40	IP-10	0.81	[0.63, 0.91]	< .001
IL-12p70	TNFα	0.91	[0.81, 0.96]	< .001
IL-12p70	substance P	0.82	[0.64, 0.91]	< .001
TNFα	substance P	0.93	[0.86, 0.97]	< .001
β endorphin	orexin A	0.81	[0.63, 0.91]	< .001
β endorphin	substance P	0.81	[0.62, 0.91]	< .001
β endorphin	αMSH	0.70	[0.44, 0.85]	0.001
orexin A	substance P	0.70	[0.44, 0.85]	0.001
orexin A	**αMSH**	0.79	[0.58, 0.90]	< .001

To assess the potential of such protein signatures in CSF to distinguish differences relating to biological variables underlying various subpopulations of CP patients, we assessed analyte correlations between various subgroups. This analysis provided novel information, with analyte signature correlations becoming apparent for specific subsets of participant groups. This report focuses on birth term as a defining characteristic; however, the Supplemental Information provides full analyses of various subgroups based on additional clinical characteristics.

### Gestational age subgroup analyses

#### Term birth

**Fig. 2:**
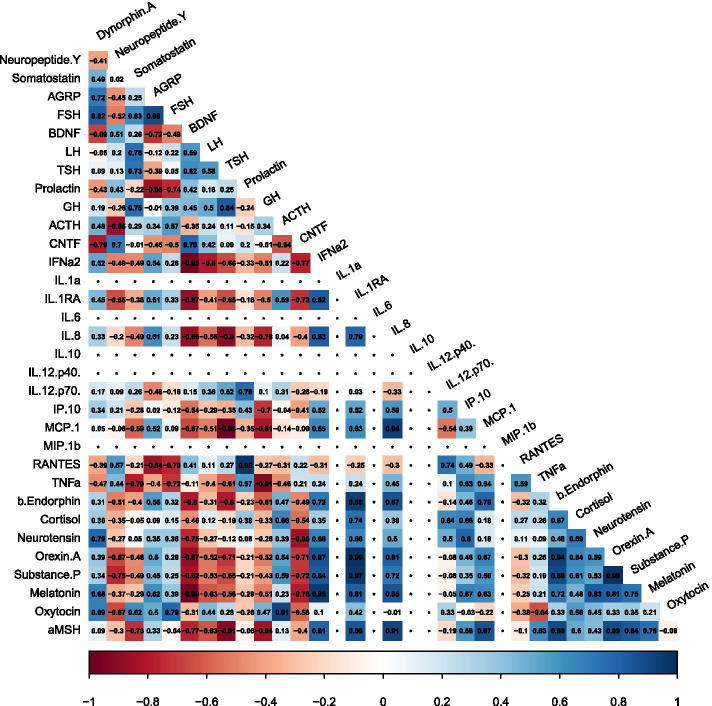
Visual representation of the direction and strength of the Pearson’s correlation coefficients between all 33 analytes assayed within the Term Birth subgroup. Positive (blue), negative (red), strong (dark shading), and weak (light shading) correlations are depicted. *In instances where the same value was reported for each variable, no correlation was calculated

No significant correlations between analytes were detected at the pre-determined significance level (*p* ≤ 0.001) used to report the rest of the subgroup findings. There were, however, two correlations at *p* ≤ 0.05 specific to Term Birth participants (correlations which were not present for Preterm Birth or Extremely Preterm Birth participants) specifically between IL-1ra and orexin A and between orexin A and substance P (identical data for both correlations: Correlation (Corr) = 0.99; 95% Confidence Interval (CI) = [0.89, 1]; Adjusted P (Adj P) = 0.041) (Fig. 2).

#### Preterm birth

**Fig. 3:**
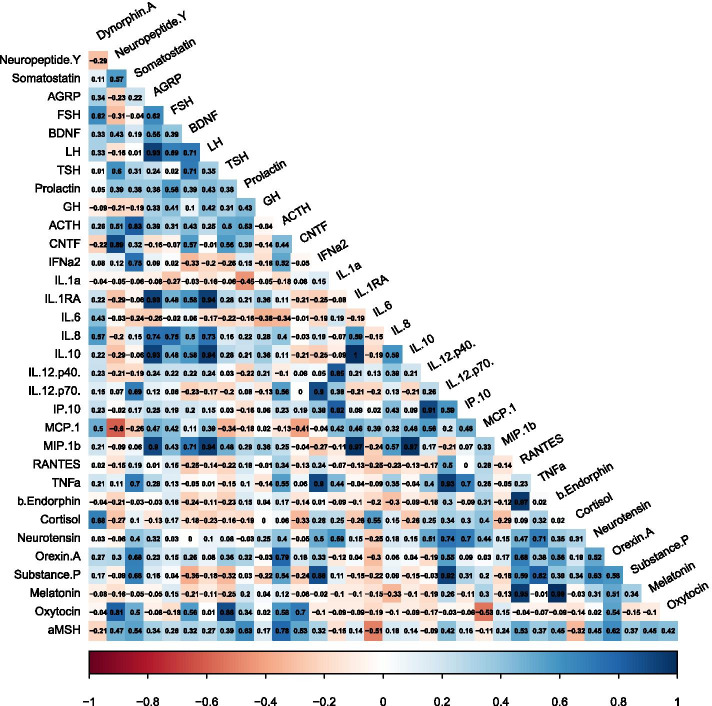
Visual representation of the direction and strength of the Pearson’s correlation coefficients between all 33 analytes assayed within the Preterm Birth subgroup. Positive (blue), negative (red), strong (dark shading), and weak (light shading) correlations are depicted

Preterm Birth status resulted in clusters of analytes that were highly correlated to one another (Fig. 3). There were 14 discrete correlations not found in the other gestational subgroup (bolded in Table 3); these correlations represent unique correlations in this sample specific to individuals born preterm.

**Table 3:** Significant Pearson’s correlations (*p* ≤ 0.001) between analyte pairs in the Preterm Birth subgroup are displayed. There were 14 unique positive analyte correlations (bold font) specific to the Preterm Birth subgroup that were not significant within the other gestational age subgroups

Analyte 1	Analyte 2	Correlation	95% CI	Adj. p
**AGRP**	**IL-1ra**	**0.93**	**[0.75, 0.98]**	**0.001**
AGRP	IL-10	0.93	[0.75, 0.98]	0.001
**IL-12.p40**	**IP-10**	**0.91**	**[0.70, 0.97]**	**0.001**
**IL-12.p70**	**substance P**	**0.92**	**[0.73, 0.98]**	**0.001**
**AGRP**	**LH**	**0.93**	**[0.77, 0.98]**	** < .001**
**LH**	**IL-1ra**	**0.94**	**[0.80, 0.98]**	** < .001**
**LH**	**IL-10**	**0.94**	**[0.80, 0.98]**	** < .001**
**LH**	**MIP-1β**	**0.94**	**[0.79, 0.98]**	** < .001**
**IL-1ra**	**IL-10**	**1.00**	**[1.00, 1.00]**	** < .001**
**IL-1ra**	**MIP-1β**	**0.97**	**[0.90, 0.99]**	** < .001**
**IL-10**	**MIP-1β**	**0.97**	**[0.90, 0.99]**	** < .001**
**IL-12.p70**	**TNFα**	**0.93**	**[0.78, 0.98]**	** < .001**
**RANTES**	**β endorphin**	**0.97**	**[0.89, 0.99]**	** < .001**
**RANTES**	**Melatonin**	**0.95**	**[0.81, 0.99]**	** < .001**
**β endorphin**	**Melatonin**	**0.99**	**[0.97, 1.00]**	** < .001**

#### Extremely preterm birth

**Fig. 4:**
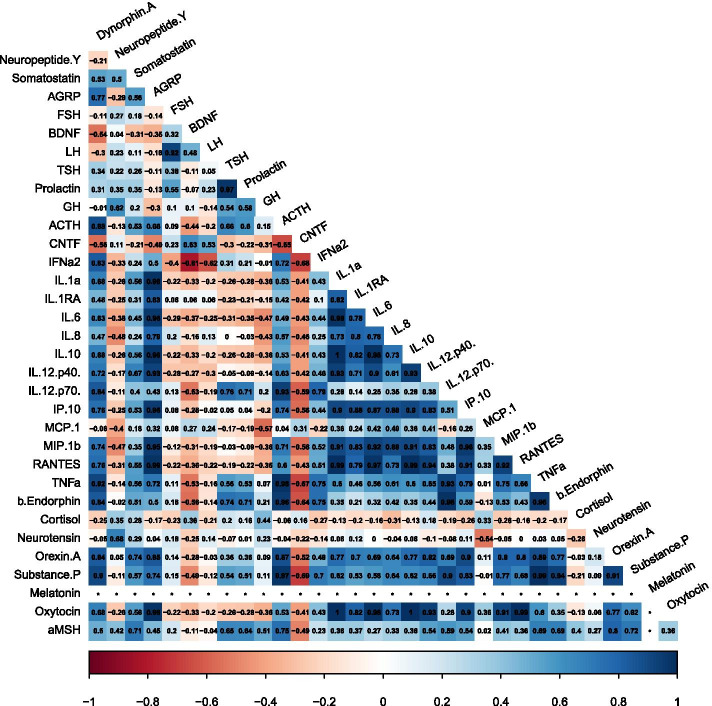
Visual representation of the direction and strength of the Pearson’s correlation coefficients between all 33 analytes assayed within the Extremely Preterm subgroup. Positive (blue), negative (red), strong (dark shading), and weak (light shading) correlations are depicted. *In instances where the same value was reported for each variable, no correlation was calculated

Extremely Preterm Birth status also resulted in distinct analyte correlations different from both Term Birth and Preterm Birth participants (Fig. 4). There were 24 discrete analyte correlations unique in this sample of participants in the extremely preterm birth status (bold in Table 4).

**Table 4:** Significant Pearson’s correlations (*p* ≤ 0.001) between analyte pairs in the Extremely Preterm Birth subgroup. There were 24 unique positive analyte correlations (bold font) specific to the Extremely Preterm Birth subgroup that were not significant within the other gestational age subgroups

Analyte 1	Analyte 2	Correlation	95% CI	Adj. p
**AGRP**	**IL-6**	**0.96**	**[0.83, 0.99]**	**0.001**
AGRP	IP-10	0.96	[0.81, 0.99]	0.001
**AGRP**	**MIP-1β**	**0.95**	**[0.79, 0.99]**	**0.001**
**ACTH**	**β endorphin**	**0.96**	**[0.81, 0.99]**	**0.001**
**IP-10**	**MIP-1β**	**0.96**	**[0.80, 0.99]**	**0.001**
**TNFα**	**β endorphin**	**0.96**	**[0.81, 0.99]**	**0.001**
**AGRP**	**IL-1α**	**0.98**	**[0.92, 1.00]**	** < .001**
**AGRP**	**IL-10**	**0.98**	**[0.92, 1.00]**	** < .001**
**AGRP**	**RANTES**	**0.99**	**[0.95, 1.00]**	** < .001**
**AGRP**	**Oxytocin**	**0.98**	**[0.92, 1.00]**	** < .001**
**TSH**	**Prolactin**	**0.97**	**[0.86, 0.99]**	** < .001**
**ACTH**	**TNFα**	**0.98**	**[0.92, 1.00]**	** < .001**
**ACTH**	**substance P**	**0.97**	**[0.88, 0.99]**	** < .001**
**IL-1α**	**IL-6**	**0.98**	**[0.92, 1.00]**	** < .001**
**IL-1α**	**IL-10**	**1.00**	**[1.00, 1.00]**	** < .001**
**IL-1α**	**RANTES**	**0.99**	**[0.96, 1.00]**	** < .001**
**IL-1α**	**Oxytocin**	**1.00**	**[1.00, 1.00]**	** < .001**
**IL-6**	**IL-10**	**0.98**	**[0.92, 1.00]**	** < .001**
**IL-6**	**RANTES**	**0.97**	**[0.87, 0.99]**	** < .001**
**IL-6**	**Oxytocin**	**0.98**	**[0.92, 1.00]**	** < .001**
**IL-10**	**RANTES**	**0.99**	**[0.96, 1.00]**	** < .001**
**IL-10**	**Oxytocin**	**1.00**	**[1.00, 1.00]**	** < .001**
**IL-12.p70**	**β endorphin**	**0.98**	**[0.92, 1.00]**	** < .001**
**RANTES**	**Oxytocin**	**0.99**	**[0.96, 1.00]**	** < .001**
**TNFα**	**substance P**	**0.99**	**[0.97, 1.00]**	** < .001**

### Between subgroup analyses

Differences in correlations between subgroups were also directly tested. As noted above, to be included in between-group analysis, each correlation first had to be statistically significant within the subgroups. Thirty-two pairs of correlations were statistically significant across all subgroups. Then the identified correlations were tested against one another to check whether they were significantly different by birth status. Based on this approach, there was a significant difference between the Preterm and Extremely Preterm Birth subgroups for the correlation between TNFα and substance P (Extremely Preterm r to z = 0.99, Preterm r to z = 0.82, Z Difference = 1.49, *p* < 0.05).

## Discussion

There are many different ways that white matter and upper motor neurons can be damaged. It is generally agreed that enough damage will interfere with and ultimately impair motor control and increase risk for the clinical condition of CP (importantly there are, of course, other associated impairments including cognitive and sensory function). Of the many putative causal agents and pathways to CP, the role of neuroinflammation in perinatal brain damage has received considerable empirical attention. There has also been considerable conceptual emphasis challenging conventional wisdom about the nature of the threat in relation to inflammation and the developing brain with a distinct shift to a perspective that a core underlying feature may be less about static events (i.e., a one-time insult) and more about sustained states (i.e., an ongoing process). The significance of this shift is in widening the focus of inquiry to include tertiary mechanisms of brain damage, which, in turn, could shed new light on a very old problem – namely treating CP based on its underlying pathophysiology.

There is a *working hypothesis* that suggests that the relationship among key neuroendocrine hormones, excitatory neuropeptides, and neuroinflammatory cytokines as markers may be a critical variable predicting outcome. The conceptual basis for this is the past two decades of work supporting the cross-talk among the endocrine, nociceptive, and immune systems. In healthy states there is an optimal balance between stress hormones and proinflammatory cytokines. In children with CP, the white matter damage may be ‘driving’ an immune-mediated inflammatory cascade. There is evidence supporting this possibility from three related studies with CP patients, one using umbilical cord serum [1], one using plasma [9], and one using blood [11].

The current analysis expands the previous work and provides further description of the molecular milieu present in the CNS of CP patients. Such documentation provides a unique opportunity to consider how differences in CNS concentrations of various inflammatory-relevant analytes between differing presentations of CP may be relevant for hypotheses about mechanisms underlying the differential outcomes in CP. Specifically, in this sample, it was observed that Term Birth, Preterm Birth, and Extremely Preterm Birth status was associated with distinct patterns of analyte inter-correlations. If reproducibility of these findings could be established, there is an opportunity to better understand the mechanisms underlying various and varying outcomes specific to birth term in CP.

The correlations present in the participants suggest relationships among systems subserving arousal (orexin A), inflammation (TNFα), anti-inflammation (IL-1ra), and neuronal excitation (substance P), with distinctions depending on birth term. Orexin A (hypocretin 1) is an excitatory, hypothalamic neuropeptide that binds to both the orexin 1 and 2 receptors (OX1R and OX2R); the former is thought to act largely through the excitatory Gq protein and both are implicated in wakefulness and sleep cycle stability [14]. Narcoleptic symptoms in dogs and mice are associated with loss-of-function mutations in the gene encoding OX2R, although immunosuppression can delay symptom onset, and deletion of the gene encoding both orexins in mice results in a full narcolepsy phenotype [14]. Substance P is another excitatory neuropeptide that binds to neurokinin 1 receptors (NK1R), which couple through the excitatory Gq protein. Substance P is contained in and released by some C-fiber nociceptors and causes activation and internalization of NK1Rs on nociceptive spinal cord neurons, exciting them [15]. These neurons project to the thalamus carrying information on the sensory-discriminative aspects of pain ultimately to the somatosensory cortex [16]. Thus, Substance P is thought to be one of the important drivers of pain transmission in spinal cord and trigeminal nucleus. IL-1ra is an endogenous anti-inflammatory cytokine that binds unproductively to the IL-1 receptor 1 (IL1R1), thereby blocking signaling by two pro-inflammatory cytokines IL-1α and IL-1β. The FDA has approved an altered form of human IL-1ra in the form of anakinra for use in peripheral inflammatory disorders like rheumatoid arthritis [17]. In the CNS, both of these cytokines, acting through IL1R1s on astrocytes, endothelial cells, and neurons, initiate transcription of multiple pro-inflammatory cytokines, including TNFα, leading to reactive gliosis and enhanced neuronal excitability following such insults as traumatic brain injury.

In clinical investigations, evidence suggests an autoimmune destruction of orexin neurons [14], and orexin A and IL-1ra in CSF have been associated with fatigue in Sjögren’s syndrome [18]. At least one study found decreased levels of substance P in the CSF of patients with narcolepsy [19], a disorder for which orexin A deficiency is a well-known biomarker [20]. Sleep dysfunction is well-established in CP; our observations suggest that specific inflammatory and nociceptive mediators may be implicated and future work should be designed to investigate in more detail the relation between sleep, inflammation, and neuronal excitation in CP based on biomarker-informed molecular signatures that may be specific to birth status.

Further, the relevance of the difference between the Preterm and Extremely Preterm subgroups appears particularly striking and significant given the literature documenting the clinical differences in phenotypic presentation between these two groups. Understanding the relationship between TNFα and substance P expression and regulation may have implications related to the underlying pathophysiology with possible prognostic and treatment relevance. One study of cultured human astrocytes demonstrated a functional interaction between these two analytes: substance P enhanced the stimulatory effect of TNFα on production of two inflammatory mediators: IL-6 and PGE_2_ [21]. This birth term-associated analyte difference may underscore the importance of our observed positive correlation to a functional interaction between a nociceptive neurotransmitter, substance P, and a pro-inflammatory cytokine, TNFα, as one of the inflammatory processes perhaps underlying the development and/or presentation of CP.

From a knowledge translation perspective, the correlation difference between Preterm and Extremely Preterm Birth subgroups for TNFα and substance P may represent a potential interaction and intervention point between inflammation and nociceptive neurotransmission specifically in the CNS; however, demonstration of a spinal or trigeminal localization of this interaction would be required to underpin this interpretation. Such an understanding may provide an opportunity to improve outcomes through earlier intervention that includes targeting these specific pathways and their unique mechanisms. One weakness of the current analysis is that the group sizes are not robust enough to distinguish larger signature patterns/differences between groups that may be informational for identifying distinctions between different patient subgroup populations; ultimately, there may be different CP patient subgroups with specific, identifiable protein signatures. Larger samples with confirmatory analyses are needed. Additionally, further consideration regarding appropriate comparison groups or control samples is warranted. Healthy controls could be useful (see below regarding a point about establishing normative values) but their use could be limited in so much as it might be more important to consider inclusion of carefully defined samples from other neurodegenerative disorders in which more is known about the underlying immune pathology and disease progression (e.g., multiple sclerosis). Doing so would provide important points of comparison for similarities and differences in inflammatory analyte levels/profiles that would, in turn, increase our understanding of inflammatory mechanisms specific to the CP phenotype.

Our long-term goal is to establish clinical value in adopting a biomarker approach to understand clinical outcomes among patients with CP. A biological marker or biomarker is any characteristic that can be measured and evaluated as an indicator of normal biologic processes, pathologic processes, or pharmacologic responses to therapeutic intervention. Biomarkers hold the potential of a better understanding of the etiology and pathology of a given disorder, providing valuable insight into diagnosis, treatment, and prognosis for many debilitating disorders and diseases. Current diagnostic and therapeutic approaches to manage chronic disability among individuals with neurodevelopmental disorders including CP are limited by our narrow understanding of the biological mechanisms underlying developmental disorders of various etiologies and confounded further by phenotypic and etiologic heterogeneity (e.g., there is not one cause of CP; clinical presentation varies widely) and the lack of biomarkers predictive of therapeutic outcome.

Part of the hope is that molecular biomarkers may provide a useful ‘work around’ for closing the gap between clinical presentation and often unknown underlying pathophysiology in CP. To get there, however, there are enormous gaps in what is known from a normative perspective within the population of CP (e.g., there are few to no referent values for expected concentration values of the vast majority of inflammatory mediators). The difficulty with any clinical sample is whether the detectable analyte represents a biomarker for ‘exposure’ (to an inflammatory process) or ‘outcome’ (of brain damage). There is no easy solution to this dilemma absent an experimental model. With that point acknowledged, we believe there is value in continuing to adopt and adapt a ‘biomarker epidemiological’ perspective as outlined by Dammann [5]. Doing so may help facilitate the development, testing, and application of immunomodulatory therapies for CP (see Fleiss and Gressens [8] for general considerations on this and related topics specific to tertiary management of brain damage as well as Lee et al. 2012 [22]).

In particular, it would be important to obtain comparison values from healthy and/or non-inflammatory based controls to establish the utility of neurochemical profile patterns as prognostic (natural history-like outcomes) or predictive tools (what profile would be most responsive to immune modulatory therapy trial). With that said, as noted above, careful selection of other well-defined diseases in which immune-mediated inflammation is a core feature would also be a valuable approach (e.g., multiple sclerosis – for which stem cell treatment trials are underway with a particular focus on patients with persistent inflammation [23]). A third approach – in a sense the strategy used for this preliminary work – is sampling within group (patient), but it would be strengthened considerably by larger samples and if/when ethically feasible, repeated measurement. Such a repeated measures approach was used by Koh et al. [24] in their investigation of cytokine changes in children with CP receiving intravenous granulocyte-colony stimulating factor followed by autologous mobilized peripheral blood mononuclear cells.

## Conclusion

Given the preliminary data presented here: 1) that endocrine, neuropeptide, and inflammatory markers are detectable in CSF from pediatric patients with CP and 2) that significant correlations exist among markers for endocrine hormones, nociceptive neuropeptides and inflammatory mediators that are distinct in various subgroups of individuals with CP, we think it is critical to continue this line of research to consider further the *functional consequences of altered inflammatory processes and responses in children with CP and to consider the potential for mechanism-driven intervention based on protein/peptide/neurotransmitter signatures in CP patients*. Increasing our scientific understanding of the neurochemical milieu involved in distinct subgroups of CP may shed light on potential targets for earlier intervention to perhaps prevent transition to more severe presentation. Clarifying a mechanistic understanding of developmental differences may be what the analyte patterns are pointing to and offer a specific starting domain for further exploration. Our hope is that these preliminary findings lay the groundwork for additional studies to confirm and expand on the potential for protein signatures in CP to be valuable clinical tools in the future.

## Supplementary Information

**Additional file 1: Table 1.** A Pearson’s correlation test against the null hypothesis of 0 was conducted for all possible pairs of analytes assayed, controlling for familywise Type I errors using the Benjamini & Hochberg (1995) false discovery rate correction. As there were 528 correlation tests, only those retaining a significant correlation (*p* ≤ 0.05) after the false discovery rate correction are presented. **Table 2.** Pearson’s correlation coefficients between pairs of analytes within the Term Birth gestational age subgroup. Only significant correlations (*p* ≤ 0.05) after controlling for false discovery rate are presented. There were no unique analyte correlations specific to those with Term Birth that were not also significant within the other gestational age subgroups. **Table 3.** Pearson’s correlation coefficients between pairs of anlytes within the Preterm Birth gestational age subgroup. Only significant correlations (*p* ≤ 0.05) after controlling for false discovery rate are presented. **Table 4.** Pearson’s correlation coefficients between pairs of analytes within the Extremely Preterm Birth gestational age subgroup. Only significant correlations (*p* ≤ 0.05) after controlling for false discovery rate are presented. **Figure 1.** Visual representation of the direction and strength of the Pearson’s correlation coefficients between analytes assayed within the subgroup with spastic CP. Positive (blue), negative (red), strong (dark shading), and weak (light shading) correlations are depicted. **Table 5A.** Pearson’s correlation coefficients between pairs of analytes within the subgroup with spastic CP. Complete dataset of significant correlations (*p* ≤ 0.05) after controlling for false discovery rate are presented. **Table 5B.** Significant Pearson’s correlations (*p* ≤ 0.001) between analyte pairs in the subgroup with spastic CP. There were 8 unique positive analyte correlations (bold font) specific to spastic CP that were not significant within the subgroup with mixed tone CP. **Figure 2.** Visual representation of the direction and strength of the Pearson’s correlation coefficients between analytes assayed within the subgroup with mixed tone CP. Positive (blue), negative (red), strong (dark shading), and weak (light shading) correlations are depicted. **Table 6A.** Pearson’s correlation coefficients between pairs of analytes within the subgroup with mixed tone CP. Complete dataset of significant correlations (*p* ≤ 0.05) after controlling for false discovery rate are presented. **Table 6B.** Significant Pearson’s correlations (*p* ≤ 0.001) between analyte pairs in the subgroup with mixed tone CP. There were 7 unique positive analyte correlations (bold font) specific to mixed tone CP that were not significant within the subgroup with spastic CP. **Figure 3.** Visual representation of the direction and strength of the Pearson’s correlation coefficients between analytes assayed within the subgroup with seizures present. Positive (blue), negative (red), strong (dark shading), and weak (light shading) correlations are depicted. **Table 7A.** Pearson’s correlation coefficients between pairs of analytes within the subgroup with seizures present. Complete dataset of significant correlations (*p* ≤ 0.05) after controlling for false discovery rate are presented. **Table 7B.** Significant Pearson’s correlations (*p* ≤ 0.001) between analyte pairs in the subgroup with seizures. There were no unique analyte correlations specific to those with seizures that were not also significant within the subgroup without seizures. **Figure 4.** Visual representation of the direction and strength of the Pearson’s correlation coefficients between analytes assayed within the subgroup without seizures. Positive (blue), negative (red), strong (dark shading), and weak (light shading) correlations are depicted. **Table 8A.** Pearson’s correlation coefficients between pairs of analytes within the subgroup without seizures. Complete dataset of significant correlations (*p* ≤ 0.05) after controlling for false discovery rate are presented. **Table 8B.** Significant Pearson’s correlations (*p* ≤ 0.001) between analyte pairs in the subgroup without seizures. There were 30 unique positive analyte correlations (bold font) specific to those without seizures that were not significant within the subgroup with seizures. **Figure 5.** Visual representation of the direction and strength of the Pearson’s correlation coefficients between analytes assayed within the subgroup with quadriplegia. Positive (blue), negative (red), strong (dark shading), and weak (light shading) correlations are depicted. **Table 9A.** Pearson’s correlation coefficients between pairs of analytes within the subgroup with quadriplegia. Complete dataset of significant correlations (*p* ≤ 0.05) after controlling for false discovery rate are presented. **Table 9B.** Significant Pearson’s correlations (*p* ≤ 0.001) between analyte pairs in the subgroup with quadriplegia. There were 16 unique positive analyte correlations (bold font) specific to those with quadriplegia that were not significant within the subgroup without quadriplegia. **Figure 6.** Visual representation of the direction and strength of the Pearson’s correlation coefficients between analytes assayed within the subgroup without quadriplegia. Positive (blue), negative (red), strong (dark shading), and weak (light shading) correlations are depicted. **Table 10A.** Pearson’s correlation coefficients between pairs of analytes within the subgroup without quadriplegia. Complete dataset of significant correlations (*p* ≤ 0.05) after controlling for false discovery rate are presented. **Table 10B.** Significant Pearson’s correlations (*p* ≤ 0.001) between analyte pairs in the subgroup without quadriplegia. There were 2 unique positive analyte correlations (bold font) specific to those without quadriplegia that were not significant within the subgroup with quadriplegia.

## Data Availability

All data generated or analyzed during this study are included in this published article [and its Supplementary information file]. The datasets analyzed are available from the corresponding author on reasonable request.

## Abbreviations

CP: Cerebral palsy
CSF: Cerebral spinal fluid
WMD: White matter damage
TNFα: Tumor necrosis factor α
mRNA: Messenger ribonucleic acid
TLR4: Toll-like receptor 4
IRB: Institutional review board
ITB: Intrathecal baclofen
ELISA: Enzyme-linked immunosorbent assay
CRL: Cytokine reference laboratory
AgRP: Agouti-related peptide
FSH: Follicle-stimulating hormone
BDNF: Brain-derived neurotrophic factor
LH: Luteinizing hormone
TSH: Thyroid stimulating hormone
GH: Growth hormone
ACTH: Adrenocorticotropic hormone
PRL: Prolactin
CNTF: Human ciliary neurotropic factor
IFNα2: Interferon alpha 2
IL-1α: Interleukin 1 alpha
IL-1ra: Interleukin 1 receptor antagonist
IL-6: Interleukin 6
IL-8: Interleukin 8
IL-10: Interleukin 10
IL-12p40: Interleukin 12 subunit 40
IL-12p70: Interleukin 12 subunit 70
IP-10: Interferon gamma-induced protein 10
MCP-1: Monocyte chemotactic protein 1
MIP1β: Macrophage inflammatory protein 1 beta
RANTES: Regulated on activation normal T expressed and secreted
TNFα: Tumor necrosis factor alpha
α-MSH: Melanocyte-stimulating hormone
DynA: Dynorphin A
PE: Phycoerythrin
Corr: Correlation
CI: Confidence Interval
Adj P: Adjusted P
M: Mean
SD: Standard Deviation
rpm: Revolutions per minute
µL: Microliters
GM-CSF: Granulocyte–macrophage colony-stimulating factor
PGE_2_: Prostaglandin E2
FDA: Food and Drug Administration
IL1R1: IL-1 receptor 1
OX1R: Orexin 1 receptor
OX2R: Orexin 2 receptor
NK1R: Neurokinin 1 receptor
Gq: Gq protein-coupled alpha subunit receptor
°C: Degrees Celsius
