# Supplementary material for: Expression of and correlational patterns among neuroinflammatory, neuropeptide, and neuroendocrine molecules from cerebrospinal fluid in cerebral palsy
Source: BMC Neurol. 2021 Oct 4;21:384. doi: 10.1186/s12883-021-02333-2 (PMC8489087; doi:10.1186/s12883-021-02333-2)
Supplement: Supplementary file 1 — Additional file 1: Table 1. A Pearson’s correlation test against the null hypothesis of 0 was conducted for all possible pairs of analytes assayed, controlling for familywise Type I errors using the Benjamini & Hochberg (1995) false discovery rate correction. As there were 528 correlation tests, only those retaining a significant correlation (p ≤ 0.05) after the false discovery rate correction are presented. Table 2. Pearson’s correlation coefficients between pairs of analytes within the Term Birth gestational age subgroup. Only significant correlations (p ≤ 0.05) after controlling for false discovery rate are presented. There were no unique analyte correlations specific to those with Term Birth that were not also significant within the other gestational age subgroups. Table 3. Pearson’s correlation coefficients between pairs of anlytes within the Preterm Birth gestational age subgroup. Only significant correlations (p ≤ 0.05) after controlling for false discovery rate are presented. Table 4. Pearson’s correlation coefficients between pairs of analytes within the Extremely Preterm Birth gestational age subgroup. Only significant correlations (p ≤ 0.05) after controlling for false discovery rate are presented. Figure 1. Visual representation of the direction and strength of the Pearson’s correlation coefficients between analytes assayed within the subgroup with spastic CP. Positive (blue), negative (red), strong (dark shading), and weak (light shading) correlations are depicted. Table 5A. Pearson’s correlation coefficients between pairs of analytes within the subgroup with spastic CP. Complete dataset of significant correlations (p ≤ 0.05) after controlling for false discovery rate are presented. Table 5B. Significant Pearson’s correlations (p ≤ 0.001) between analyte pairs in the subgroup with spastic CP. There were 8 unique positive analyte correlations (bold font) specific to spastic CP that were not significant within the subgroup with mixed tone CP. Figure 2. Vi [file 12883_2021_2333_MOESM1_ESM.docx]

**Supplemental Data**

Table 1. A Pearson’s correlation test against the null hypothesis of 0 was conducted for all possible pairs of analytes assayed, controlling for familywise Type I errors using the Benjamini & Hochberg (1995) false discovery rate correction. As there were 528 correlation tests, only those retaining a significant correlation (p ≤ 0.05) after the false discovery rate correction are presented.

| **Analyte 1** | **Analyte 2** | **Correlation** | **95% CI** | **Adj. p** |
| --- | --- | --- | --- | --- |
| Dynorphin A | AGRP | 0.71 | [0.45, 0.85] | < .001 |
| Dynorphin A | ACTH | 0.79 | [0.60, 0.90] | < .001 |
| Dynorphin A | IL-6 | 0.59 | [0.28, 0.79] | 0.008 |
| Dynorphin A | IL-8 | 0.50 | [0.15, 0.73] | 0.043 |
| Dynorphin A | IL-10 | 0.63 | [0.34, 0.81] | 0.004 |
| Dynorphin A | IL-12.p40 | 0.60 | [0.29, 0.80] | 0.007 |
| Dynorphin A | IL-12.p70 | 0.68 | [0.41, 0.84] | 0.001 |
| Dynorphin A | IP-10 | 0.51 | [0.17, 0.74] | 0.036 |
| Dynorphin A | MIP-1β | 0.64 | [0.35, 0.82] | 0.003 |
| Dynorphin A | TNFα | 0.80 | [0.60, 0.90] | < .001 |
| Dynorphin A | β endorphin | 0.55 | [0.23, 0.77] | 0.016 |
| Dynorphin A | substance P | 0.78 | [0.57, 0.89] | < .001 |
| Neuropeptide Y | CNTF | 0.59 | [0.28, 0.79] | 0.008 |
| AGRP | ACTH | 0.65 | [0.37, 0.82] | 0.002 |
| AGRP | IL-1ra | 0.56 | [0.23, 0.77] | 0.016 |
| AGRP | IL-6 | 0.73 | [0.49, 0.87] | < .001 |
| AGRP | IL-8 | 0.71 | [0.46, 0.86] | < .001 |
| AGRP | IL-10 | 0.91 | [0.81, 0.96] | < .001 |
| AGRP | IL-12.p40 | 0.73 | [0.50, 0.87] | < .001 |
| AGRP | IP-10 | 0.62 | [0.32, 0.81] | 0.005 |
| AGRP | MIP-1β | 0.94 | [0.87, 0.97] | < .001 |
| AGRP | TNFα | 0.67 | [0.40, 0.84] | 0.001 |
| AGRP | substance P | 0.66 | [0.38, 0.83] | 0.002 |
| FSH | LH | 0.89 | [0.78, 0.95] | < .001 |
| BDNF | CNTF | 0.61 | [0.30, 0.80] | 0.007 |
| TSH | ACTH | 0.69 | [0.42, 0.84] | 0.001 |
| TSH | IL-12.p70 | 0.59 | [0.28, 0.79] | 0.008 |
| TSH | TNFα | 0.53 | [0.20, 0.75] | 0.025 |
| TSH | β endorphin | 0.49 | [0.14, 0.73] | 0.047 |
| TSH | substance P | 0.50 | [0.16, 0.74] | 0.041 |
| ACTH | IL-8 | 0.54 | [0.21, 0.76] | 0.022 |
| ACTH | IL-10 | 0.54 | [0.21, 0.76] | 0.020 |
| ACTH | IL-12.p40 | 0.49 | [0.15, 0.73] | 0.045 |
| ACTH | IL-12.p70 | 0.82 | [0.65, 0.92] | < .001 |
| ACTH | MIP-1β | 0.66 | [0.39, 0.83] | 0.002 |
| ACTH | TNFα | 0.92 | [0.83, 0.96] | < .001 |
| ACTH | β endorphin | 0.67 | [0.40, 0.83] | 0.001 |
| ACTH | Orexin A | 0.53 | [0.20, 0.76] | 0.023 |
| ACTH | substance P | 0.91 | [0.81, 0.96] | < .001 |
| ACTH | αMSH | 0.56 | [0.23, 0.77] | 0.015 |
| IFNα2 | IL-12.p70 | 0.73 | [0.49, 0.87] | < .001 |
| IFNα2 | TNFα | 0.63 | [0.34, 0.81] | 0.004 |
| IFNα2 | substance P | 0.56 | [0.24, 0.77] | 0.014 |
| IL-1α | IL-12.p40 | 0.78 | [0.57, 0.89] | < .001 |
| IL-1α | IP-10 | 0.79 | [0.60, 0.90] | < .001 |
| IL-1ra | IL-8 | 0.66 | [0.38, 0.83] | 0.002 |
| IL-1ra | MIP-1β | 0.55 | [0.23, 0.77] | 0.017 |
| IL-1ra | β endorphin | 0.51 | [0.16, 0.74] | 0.038 |
| IL-1ra | Orexin A | 0.75 | [0.52, 0.88] | < .001 |
| IL-1ra | Melatonin | 0.56 | [0.24, 0.77] | 0.014 |
| IL-1ra | αMSH | 0.52 | [0.18, 0.75] | 0.030 |
| IL-6 | IL-8 | 0.50 | [0.16, 0.74] | 0.041 |
| IL-6 | IL-10 | 0.87 | [0.74, 0.94] | < .001 |
| IL-6 | IL-12.p40 | 0.68 | [0.41, 0.84] | 0.001 |
| IL-6 | IP-10 | 0.49 | [0.14, 0.73] | 0.050 |
| IL-6 | MIP-1β | 0.66 | [0.38, 0.83] | 0.002 |
| IL-6 | TNFα | 0.49 | [0.14, 0.73] | 0.050 |
| IL-8 | IL-10 | 0.60 | [0.29, 0.80] | 0.007 |
| IL-8 | IL-12.p40 | 0.50 | [0.15, 0.73] | 0.042 |
| IL-8 | IP-10 | 0.58 | [0.27, 0.78] | 0.010 |
| IL-8 | MCP-1 | 0.56 | [0.24, 0.77] | 0.014 |
| IL-8 | MIP-1β | 0.73 | [0.49, 0.87] | < .001 |
| IL-8 | TNFα | 0.54 | [0.21, 0.76] | 0.022 |
| IL-8 | β endorphin | 0.48 | [0.14, 0.73] | 0.050 |
| IL-8 | Orexin A | 0.59 | [0.28, 0.79] | 0.008 |
| IL-8 | substance P | 0.60 | [0.29, 0.79] | 0.008 |
| IL-8 | αMSH | 0.50 | [0.16, 0.74] | 0.041 |
| IL-10 | IL-12.p40 | 0.74 | [0.51, 0.87] | < .001 |
| IL-10 | IP-10 | 0.57 | [0.25, 0.78] | 0.014 |
| IL-10 | MIP-1β | 0.81 | [0.63, 0.91] | < .001 |
| IL-10 | TNFα | 0.58 | [0.26, 0.78] | 0.010 |
| IL-10 | substance P | 0.60 | [0.30, 0.80] | 0.007 |
| IL-12.p40 | IP-10 | 0.81 | [0.63, 0.91] | < .001 |
| IL-12.p40 | MCP-1 | 0.48 | [0.14, 0.73] | 0.050 |
| IL-12.p40 | MIP-1β | 0.63 | [0.34, 0.81] | 0.003 |
| IL-12.p40 | TNFα | 0.60 | [0.29, 0.79] | 0.007 |
| IL-12.p40 | substance P | 0.49 | [0.14, 0.73] | 0.047 |
| IL-12.p70 | IP-10 | 0.50 | [0.16, 0.74] | 0.041 |
| IL-12.p70 | TNFα | 0.91 | [0.81, 0.96] | < .001 |
| IL-12.p70 | β endorphin | 0.59 | [0.28, 0.79] | 0.008 |
| IL-12.p70 | substance P | 0.82 | [0.64, 0.91] | < .001 |
| IP-10 | MIP-1β | 0.52 | [0.18, 0.75] | 0.031 |
| IP-10 | TNFα | 0.65 | [0.36, 0.82] | 0.002 |
| IP-10 | substance P | 0.55 | [0.22, 0.77] | 0.017 |
| MIP-1β | TNFα | 0.61 | [0.31, 0.80] | 0.006 |
| MIP-1β | substance P | 0.61 | [0.30, 0.80] | 0.006 |
| TNFα | β endorphin | 0.64 | [0.35, 0.82] | 0.003 |
| TNFα | substance P | 0.93 | [0.86, 0.97] | < .001 |
| β endorphin | Orexin. A | 0.81 | [0.63, 0.91] | < .001 |
| β endorphin | substance P | 0.81 | [0.62, 0.91] | < .001 |
| β endorphin | αMSH | 0.70 | [0.44, 0.85] | 0.001 |
| Neurotensin | Melatonin | 0.49 | [0.14, 0.73] | 0.050 |
| Orexin A | substance P | 0.70 | [0.44, 0.85] | 0.001 |
| Orexin A | Melatonin | 0.60 | [0.29, 0.79] | 0.007 |
| Orexin A | αMSH | 0.79 | [0.58, 0.90] | < .001 |
| substance P | αMSH | 0.59 | [0.28, 0.79] | 0.008 |

Table 2. Pearson’s correlation coefficients between pairs of analytes within the Term Birth gestational age subgroup. Only significant correlations (p ≤ 0.05) after controlling for false discovery rate are presented. There were no unique analyte correlations specific to those with Term Birth that were not also significant within the other gestational age subgroups.

| **Analyte 1** | **Analyte 2** | **Correlation** | **CI** | **Adj. p** |
| --- | --- | --- | --- | --- |
| IL-1ra | Orexin A | 0.99 | [0.89, 1.00] | 0.041 |
| Orexin A | substance P | 0.99 | [0.89, 1.00] | 0.041 |

Table 3. Pearson’s correlation coefficients between pairs of anlytes within the Preterm Birth gestational age subgroup. Only significant correlations (p ≤ 0.05) after controlling for false discovery rate are presented.

| **Analyte 1** | **Analyte 2** | **Correlation** | **95% CI** | **Adj. p** |
| --- | --- | --- | --- | --- |
| Neuropeptide Y | CNTF | 0.89 | [0.65, 0.97] | 0.003 |
| Neuropeptide Y | Oxytocin | 0.81 | [0.45, 0.95] | 0.027 |
| Somatostatin | ACTH | 0.83 | [0.49, 0.95] | 0.020 |
| AGRP | LH | 0.93 | [0.77, 0.98] | < .001 |
| AGRP | IL-1ra | 0.93 | [0.75, 0.98] | 0.001 |
| AGRP | IL-10 | 0.93 | [0.75, 0.98] | 0.001 |
| AGRP | MIP-1β | 0.90 | [0.68, 0.97] | 0.002 |
| LH | IL-1ra | 0.94 | [0.80, 0.98] | < .001 |
| LH | IL-10 | 0.94 | [0.80, 0.98] | < .001 |
| LH | MIP-1β | 0.94 | [0.79, 0.98] | < .001 |
| TSH | Oxytocin | 0.88 | [0.62, 0.97] | 0.004 |
| ACTH | Orexin A | 0.79 | [0.39, 0.94] | 0.048 |
| ACTH | αMSH | 0.78 | [0.38, 0.94] | 0.050 |
| IFNα2 | IL-12.p70 | 0.90 | [0.67, 0.97] | 0.002 |
| IFNα2 | TNFα | 0.90 | [0.68, 0.97] | 0.002 |
| IFNα2 | substance P | 0.88 | [0.62, 0.97] | 0.004 |
| IL-1α | IL-12.p40 | 0.85 | [0.53, 0.96] | 0.012 |
| IL-1α | IP-10 | 0.82 | [0.48, 0.95] | 0.021 |
| IL-1ra | IL-10 | 1.00 | [1.00, 1.00] | < .001 |
| IL-1ra | MIP-1β | 0.97 | [0.90, 0.99] | < .001 |
| IL-10 | MIP-1β | 0.97 | [0.90, 0.99] | < .001 |
| IL-12.p40 | IP-10 | 0.91 | [0.70, 0.97] | 0.001 |
| IL-12.p70 | TNFα | 0.93 | [0.78, 0.98] | < .001 |
| IL-12.p70 | substance P | 0.92 | [0.73, 0.98] | 0.001 |
| RANTES | β endorphin | 0.97 | [0.89, 0.99] | < .001 |
| RANTES | Melatonin | 0.95 | [0.81, 0.99] | < .001 |
| TNFα | substance P | 0.82 | [0.46, 0.95] | 0.024 |
| β endorphin | Melatonin | 0.99 | [0.97, 1.00] | < .001 |

Table 4. Pearson’s correlation coefficients between pairs of analytes within the Extremely Preterm Birth gestational age subgroup. Only significant correlations (p ≤ 0.05) after controlling for false discovery rate are presented.

| **Analyte 1** | **Analyte 2** | **Correlation** | **95% CI** | **Adj. p** |
| --- | --- | --- | --- | --- |
| Dynorphin A | ACTH | 0.88 | [0.52, 0.97] | 0.017 |
| Dynorphin A | IFNα2 | 0.83 | [0.38, 0.96] | 0.042 |
| Dynorphin A | IL-12.p70 | 0.84 | [0.41, 0.97] | 0.036 |
| Dynorphin A | TNFα | 0.92 | [0.65, 0.98] | 0.007 |
| Dynorphin A | β endorphin | 0.84 | [0.40, 0.97] | 0.038 |
| Dynorphin A | Orexin A | 0.84 | [0.40, 0.97] | 0.039 |
| Dynorphin A | substance P | 0.90 | [0.60, 0.98] | 0.009 |
| AGRP | IL-1α | 0.98 | [0.92, 1.00] | < .001 |
| AGRP | IL-1ra | 0.83 | [0.38, 0.96] | 0.042 |
| AGRP | IL-6 | 0.96 | [0.83, 0.99] | 0.001 |
| AGRP | IL-10 | 0.98 | [0.92, 1.00] | < .001 |
| AGRP | IL-12.p40 | 0.93 | [0.69, 0.98] | 0.005 |
| AGRP | IP-10 | 0.96 | [0.81, 0.99] | 0.001 |
| AGRP | MIP-1β | 0.95 | [0.79, 0.99] | 0.001 |
| AGRP | RANTES | 0.99 | [0.95, 1.00] | < .001 |
| AGRP | Orexin A | 0.85 | [0.42, 0.97] | 0.033 |
| AGRP | Oxytocin | 0.98 | [0.92, 1.00] | < .001 |
| FSH | LH | 0.92 | [0.66, 0.98] | 0.006 |
| TSH | Prolactin | 0.97 | [0.86, 0.99] | < .001 |
| ACTH | IL-12.p70 | 0.93 | [0.71, 0.99] | 0.004 |
| ACTH | TNFα | 0.98 | [0.92, 1.00] | < .001 |
| ACTH | β endorphin | 0.96 | [0.81, 0.99] | 0.001 |
| ACTH | Orexin A | 0.87 | [0.49, 0.97] | 0.020 |
| ACTH | substance P | 0.97 | [0.88, 0.99] | < .001 |
| IL-1α | IL-6 | 0.98 | [0.92, 1.00] | < .001 |
| IL-1α | IL-10 | 1.00 | [1.00, 1.00] | < .001 |
| IL-1α | IL-12.p40 | 0.93 | [0.71, 0.99] | 0.004 |
| IL-1α | IP-10 | 0.90 | [0.59, 0.98] | 0.010 |
| IL-1α | MIP-1β | 0.91 | [0.60, 0.98] | 0.009 |
| IL-1α | RANTES | 0.99 | [0.96, 1.00] | < .001 |
| IL-1α | Oxytocin | 1.00 | [1.00, 1.00] | < .001 |
| IL-1ra | IP-10 | 0.88 | [0.54, 0.98] | 0.014 |
| IL-1ra | MIP-1β | 0.83 | [0.38, 0.96] | 0.042 |
| IL-6 | IL-10 | 0.98 | [0.92, 1.00] | < .001 |
| IL-6 | IL-12.p40 | 0.90 | [0.57, 0.98] | 0.011 |
| IL-6 | IP-10 | 0.87 | [0.5, 0.97] | 0.019 |
| IL-6 | MIP-1β | 0.92 | [0.67, 0.98] | 0.006 |
| IL-6 | RANTES | 0.97 | [0.87, 0.99] | < .001 |
| IL-6 | Oxytocin | 0.98 | [0.92, 1.00] | < .001 |
| IL-8 | IP-10 | 0.88 | [0.51, 0.97] | 0.018 |
| IL-8 | MIP-1β | 0.89 | [0.57, 0.98] | 0.011 |
| IL-10 | IL-12.p40 | 0.93 | [0.71, 0.99] | 0.004 |
| IL-10 | IP-10 | 0.90 | [0.59, 0.98] | 0.010 |
| IL-10 | MIP-1β | 0.91 | [0.6, 0.98] | 0.009 |
| IL-10 | RANTES | 0.99 | [0.96, 1.00] | < .001 |
| IL-10 | Oxytocin | 1.00 | [1.00, 1.00] | < .001 |
| IL-12.p40 | IP-10 | 0.83 | [0.37, 0.96] | 0.042 |
| IL-12.p40 | MIP-1β | 0.83 | [0.38, 0.96] | 0.042 |
| IL-12.p40 | RANTES | 0.94 | [0.73, 0.99] | 0.003 |
| IL-12.p40 | Oxytocin | 0.93 | [0.71, 0.99] | 0.004 |
| IL-12.p70 | TNFα | 0.93 | [0.68, 0.98] | 0.005 |
| IL-12.p70 | β endorphin | 0.98 | [0.92, 1.00] | < .001 |
| IL-12.p70 | substance P | 0.90 | [0.57, 0.98] | 0.011 |
| IP-10 | MIP.1b | 0.96 | [0.80, 0.99] | 0.001 |
| IP-10 | RANTES | 0.91 | [0.62, 0.98] | 0.008 |
| IP-10 | Orexin A | 0.90 | [0.58, 0.98] | 0.011 |
| IP-10 | substance P | 0.83 | [0.37, 0.96] | 0.043 |
| IP-10 | Oxytocin | 0.90 | [0.59, 0.98] | 0.010 |
| MIP-1β | RANTES | 0.92 | [0.66, 0.98] | 0.006 |
| MIP-1β | Oxytocin | 0.91 | [0.60, 0.98] | 0.009 |
| RANTES | Oxytocin | 0.99 | [0.96, 1.00] | < .001 |
| TNFα | β endorphin | 0.96 | [0.81, 0.99] | 0.001 |
| TNFα | Orexin A | 0.89 | [0.54, 0.98] | 0.014 |
| TNFα | substance P | 0.99 | [0.97, 1.00] | < .001 |
| β endorphin | substance P | 0.94 | [0.73, 0.99] | 0.003 |
| Orexin A | substance P | 0.91 | [0.64, 0.98] | 0.007 |


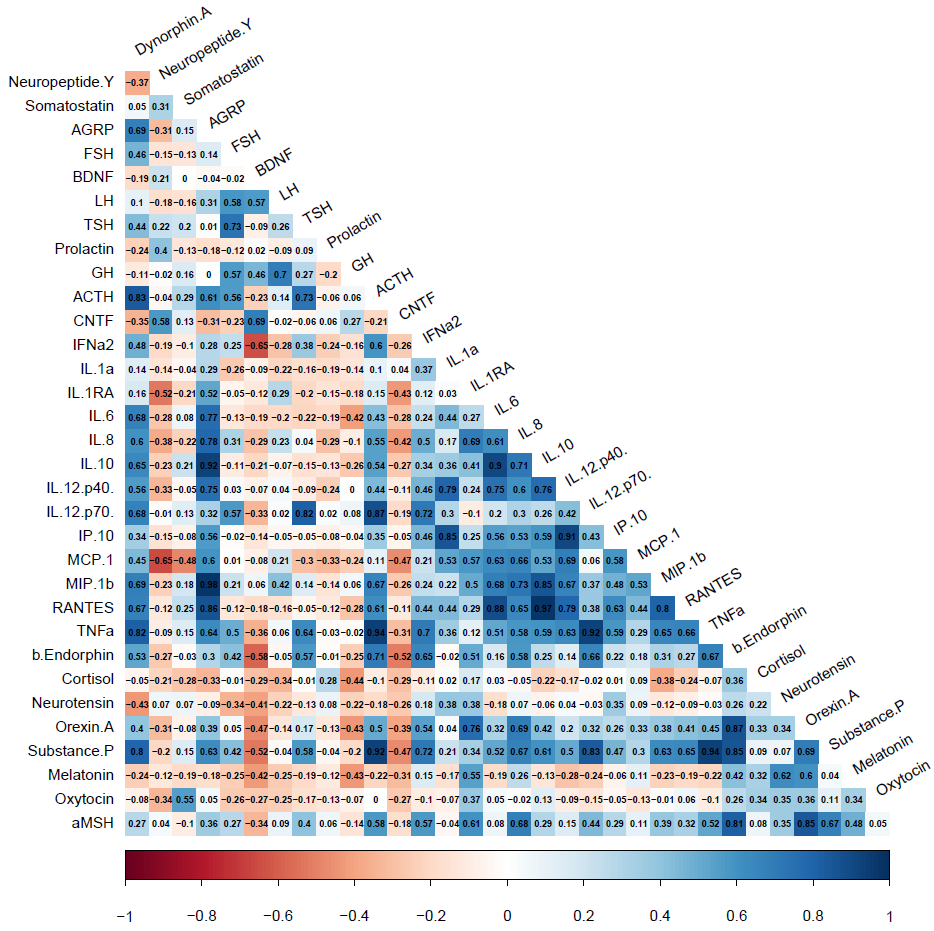


Figure 1. Visual representation of the direction and strength of the Pearson’s correlation coefficients between analytes assayed within the subgroup with spastic CP. Positive (blue), negative (red), strong (dark shading), and weak (light shading) correlations are depicted.

Table 5A. Pearson’s correlation coefficients between pairs of analytes within the subgroup with spastic CP. Complete dataset of significant correlations (p ≤ 0.05) after controlling for false discovery rate are presented.

| **Analyte 1** | **Analyte 2** | **Correlation** | **95% CI** | **Adj. p** |
| --- | --- | --- | --- | --- |
| Dynorphin A | ACTH | 0.83 | [0.51, 0.95] | 0.012 |
| Dynorphin A | TNFα | 0.82 | [0.48, 0.94] | 0.017 |
| Dynorphin A | substance P | 0.80 | [0.44, 0.94] | 0.023 |
| AGRP | IL-6 | 0.77 | [0.38, 0.93] | 0.039 |
| AGRP | IL-8 | 0.78 | [0.41, 0.93] | 0.031 |
| AGRP | IL-10 | 0.92 | [0.75, 0.98] | 0.001 |
| AGRP | MIP-1β | 0.98 | [0.92, 0.99] | < .001 |
| AGRP | RANTES | 0.86 | [0.60, 0.96] | 0.006 |
| TSH | IL-12.p70 | 0.82 | [0.49, 0.94] | 0.017 |
| ACTH | IL-12.p70 | 0.87 | [0.61, 0.96] | 0.005 |
| ACTH | TNFα | 0.94 | [0.80, 0.98] | < .001 |
| ACTH | substance P | 0.92 | [0.75, 0.98] | 0.001 |
| IL-1α | IL-12.p40 | 0.79 | [0.43, 0.93] | 0.027 |
| IL-1α | IP-10 | 0.85 | [0.57, 0.96] | 0.007 |
| IL-1ra | Orexin A | 0.76 | [0.37, 0.93] | 0.043 |
| IL-6 | IL-10 | 0.90 | [0.70, 0.97] | 0.001 |
| IL-6 | RANTES | 0.88 | [0.64, 0.96] | 0.004 |
| IL-10 | IL-12.p40 | 0.76 | [0.36, 0.92] | 0.043 |
| IL-10 | MIP-1β | 0.85 | [0.56, 0.95] | 0.008 |
| IL-10 | RANTES | 0.97 | [0.90, 0.99] | < .001 |
| IL-12.p40 | IP-10 | 0.91 | [0.71, 0.97] | 0.001 |
| IL-12.p40 | RANTES | 0.79 | [0.42, 0.93] | 0.028 |
| IL-12.p70 | TNFα | 0.92 | [0.75, 0.98] | 0.001 |
| IL-12.p70 | substance P | 0.83 | [0.52, 0.95] | 0.012 |
| TNFα | substance P | 0.94 | [0.80, 0.98] | < .001 |
| β endorphin | Orexin A | 0.87 | [0.61, 0.96] | 0.005 |
| β endorphin | substance P | 0.85 | [0.57, 0.96] | 0.007 |
| β endorphin | αMSH | 0.81 | [0.47, 0.94] | 0.018 |
| Orexin A | αMSH | 0.85 | [0.55, 0.95] | 0.009 |

Table 5B. Significant Pearson’s correlations (p ≤ 0.001) between analyte pairs in the subgroup with spastic CP. There were 8 unique positive analyte correlations (bold font) specific to spastic CP that were not significant within the subgroup with mixed tone CP.

| **Analyte 1** | **Analyte 2** | **Correlation** | **CI** | **Adj. P** |
| --- | --- | --- | --- | --- |
| **AGRP** | **IL-10** | **0.92** | **[0.75, 0.98]** | **0.001** |
| **ACTH** | **substance P** | **0.92** | **[0.75, 0.98]** | **0.001** |
| **IL-6** | **IL-10** | **0.90** | **[0.70, 0.97]** | **0.001** |
| **IL-12.p40** | **IP-10** | **0.91** | **[0.71, 0.97]** | **0.001** |
| IL-12.p70 | TNFα | 0.92 | [0.75, 0.98] | 0.001 |
| **AGRP** | **MIP-1β** | **0.98** | **[0.92, 0.99]** | **< .001** |
| **ACTH** | **TNFα** | **0.94** | **[0.80, 0.98]** | **< .001** |
| **IL-10** | **RANTES** | **0.97** | **[0.90, 0.99]** | **< .001** |
| **TNFα** | **substance P** | **0.94** | **[0.80, 0.98]** | **< .001** |


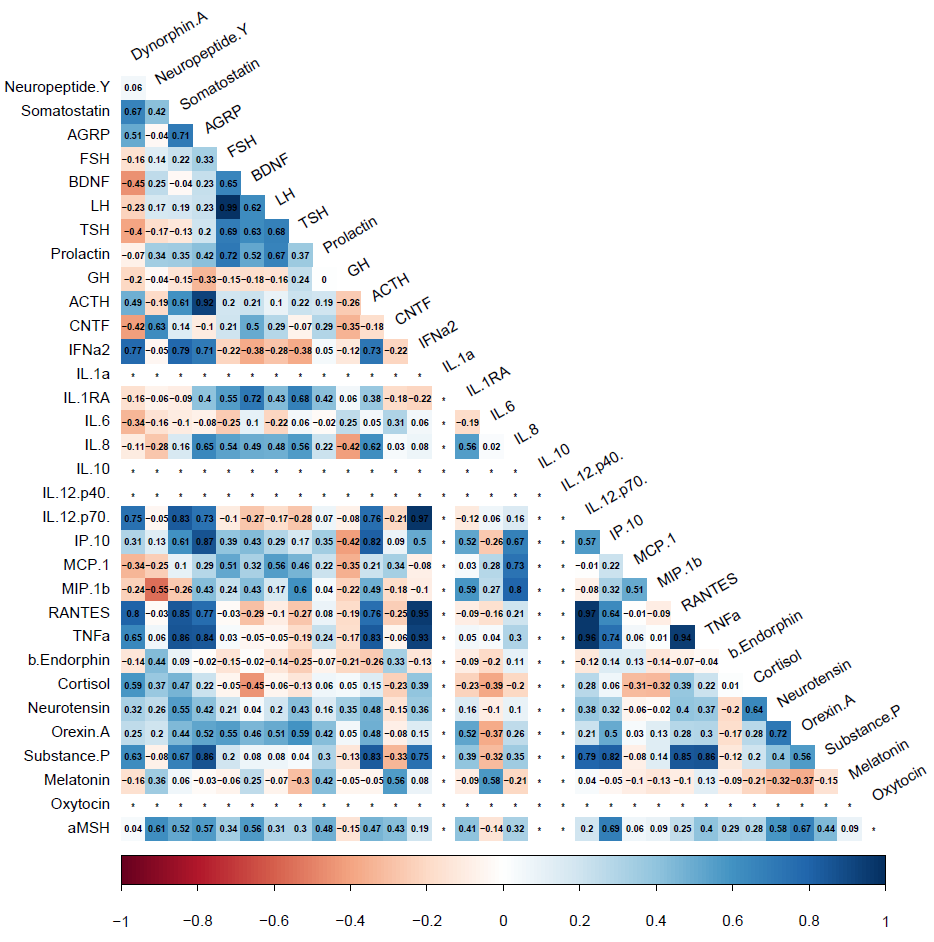


Figure 2. Visual representation of the direction and strength of the Pearson’s correlation coefficients between analytes assayed within the subgroup with mixed tone CP. Positive (blue), negative (red), strong (dark shading), and weak (light shading) correlations are depicted.

Table 6A: Pearson’s correlation coefficients between pairs of analytes within the subgroup with mixed tone CP. Complete dataset of significant correlations (p ≤ 0.05) after controlling for false discovery rate are presented.

| **Analyte 1** | **Analyte 2** | **Correlation** | **95% CI** | **Adj. p** |
| --- | --- | --- | --- | --- |
| Dynorphin A | RANTES | 0.80 | [0.42, 0.94] | 0.034 |
| Somatostatin | IFNα2 | 0.79 | [0.38, 0.94] | 0.042 |
| Somatostatin | IL-12.p70 | 0.83 | [0.49, 0.95] | 0.019 |
| Somatostatin | RANTES | 0.85 | [0.53, 0.96] | 0.016 |
| Somatostatin | TNFα | 0.86 | [0.56, 0.96] | 0.012 |
| AGRP | ACTH | 0.92 | [0.75, 0.98] | 0.001 |
| AGRP | IP-10 | 0.87 | [0.58, 0.96] | 0.012 |
| AGRP | TNFα | 0.84 | [0.52, 0.95] | 0.016 |
| AGRP | substance P | 0.86 | [0.57, 0.96] | 0.012 |
| FSH | LH | 0.99 | [0.95, 1.00] | < .001 |
| ACTH | IP-10 | 0.82 | [0.46, 0.95] | 0.024 |
| ACTH | TNFα | 0.83 | [0.49, 0.95] | 0.019 |
| ACTH | substance P | 0.83 | [0.49, 0.95] | 0.019 |
| IFNα2 | IL-12.p70 | 0.97 | [0.90, 0.99] | < .001 |
| IFNα2 | RANTES | 0.95 | [0.84, 0.99] | < .001 |
| IFNα2 | TNFα | 0.93 | [0.75, 0.98] | 0.001 |
| IL-8 | MIP-1β | 0.80 | [0.41, 0.94] | 0.035 |
| IL-12.p70 | RANTES | 0.97 | [0.89, 0.99] | < .001 |
| IL-12.p70 | TNFα | 0.96 | [0.87, 0.99] | < .001 |
| IL-12.p70 | substance P | 0.79 | [0.40, 0.94] | 0.038 |
| IP-10 | substance P | 0.82 | [0.46, 0.95] | 0.024 |
| RANTES | TNFα | 0.94 | [0.81, 0.98] | < .001 |
| RANTES | substance P | 0.85 | [0.53, 0.96] | 0.016 |
| TNFα | substance P | 0.86 | [0.56, 0.96] | 0.012 |

Table 6B. Significant Pearson’s correlations (p ≤ 0.001) between analyte pairs in the subgroup with mixed tone CP. There were 7 unique positive analyte correlations (bold font) specific to mixed tone CP that were not significant within the subgroup with spastic CP.

| **Analyte 1** | **Analyte 2** | **Correlation** | **CI** | **Adj. p** |
| --- | --- | --- | --- | --- |
| **AGRP** | **ACTH** | **0.92** | **[0.75, 0.98]** | **0.001** |
| **IFNα2** | **TNFα** | **0.93** | **[0.75, 0.98]** | **0.001** |
| **FSH** | **LH** | **0.99** | **[0.95, 1.00]** | **< .001** |
| **IFNα2** | **IL-12.p70** | **0.97** | **[0.9, 0.99]** | **< .001** |
| **IFNα2** | **RANTES** | **0.95** | **[0.84, 0.99]** | **< .001** |
| **IL-12.p70** | **RANTES** | **0.97** | **[0.89, 0.99]** | **< .001** |
| IL-12.p70 | TNFα | 0.96 | [0.87, 0.99] | < .001 |
| **RANTES** | **TNFα** | **0.94** | **[0.81, 0.98]** | **< .001** |


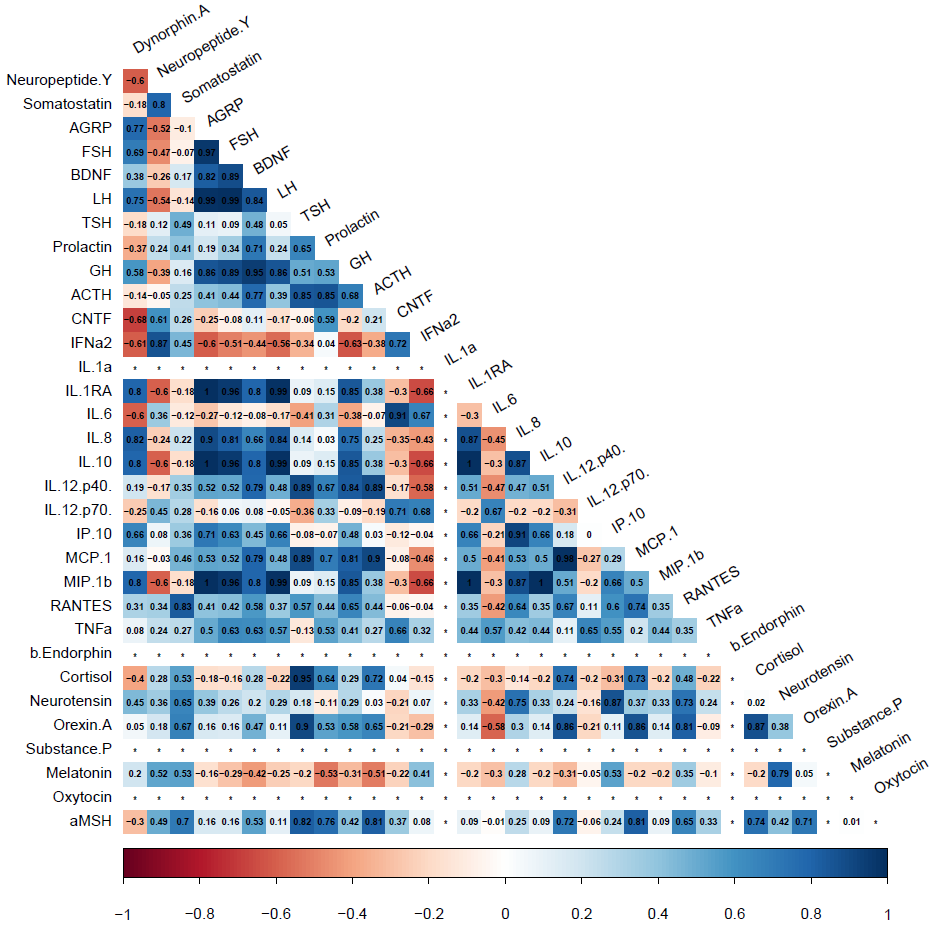


Figure 3. Visual representation of the direction and strength of the Pearson’s correlation coefficients between analytes assayed within the subgroup with seizures present. Positive (blue), negative (red), strong (dark shading), and weak (light shading) correlations are depicted.

Table 7A: Pearson’s correlation coefficients between pairs of analytes within the subgroup with seizures present. Complete dataset of significant correlations (p ≤ 0.05) after controlling for false discovery rate are presented.

| **Analyte 1** | **Analyte 2** | **Correlation** | **CI** | **Adj P** |
| --- | --- | --- | --- | --- |
| AGRP | LH | 0.99 | [0.90, 1.00] | 0.009 |
| AGRP | IL-1ra | 1.00 | [0.96, 1.00] | 0.002 |
| AGRP | IL-10 | 1.00 | [0.96, 1.00] | 0.002 |
| AGRP | MIP-1β | 1.00 | [0.96, 1.00] | 0.002 |
| FSH | LH | 0.99 | [0.91, 1.00] | 0.008 |
| LH | IL-1ra | 0.99 | [0.88, 1.00] | 0.009 |
| LH | IL-10 | 0.99 | [0.88, 1.00] | 0.009 |
| LH | MIP-1β | 0.99 | [0.88, 1.00] | 0.009 |
| IL-1ra | IL-10 | 1.00 | [1.00, 1.00] | < .001 |
| IL-1ra | MIP-1β | 1.00 | [1.00, 1.00] | < .001 |
| IL-10 | MIP-1β | 1.00 | [1.00, 1.00] | < .001 |
| IL-12.p40 | MCP-1 | 0.98 | [0.85, 1.00] | 0.015 |

Table 7B. Significant Pearson’s correlations (p ≤ 0.001) between analyte pairs in the subgroup with seizures. There were no unique analyte correlations specific to those with seizures that were not also significant within the subgroup without seizures.

| **Analyte 1** | **Analyte 2** | **Correlation** | **CI** | **Adj. p** |
| --- | --- | --- | --- | --- |
| IL-1ra | IL-10 | 1.00 | [1.00, 1.00] | < .001 |
| IL-1ra | MIP-1β | 1.00 | [1.00, 1.00] | < .001 |
| IL-10 | MIP-1β | 1.00 | [1.00, 1.00] | < .001 |


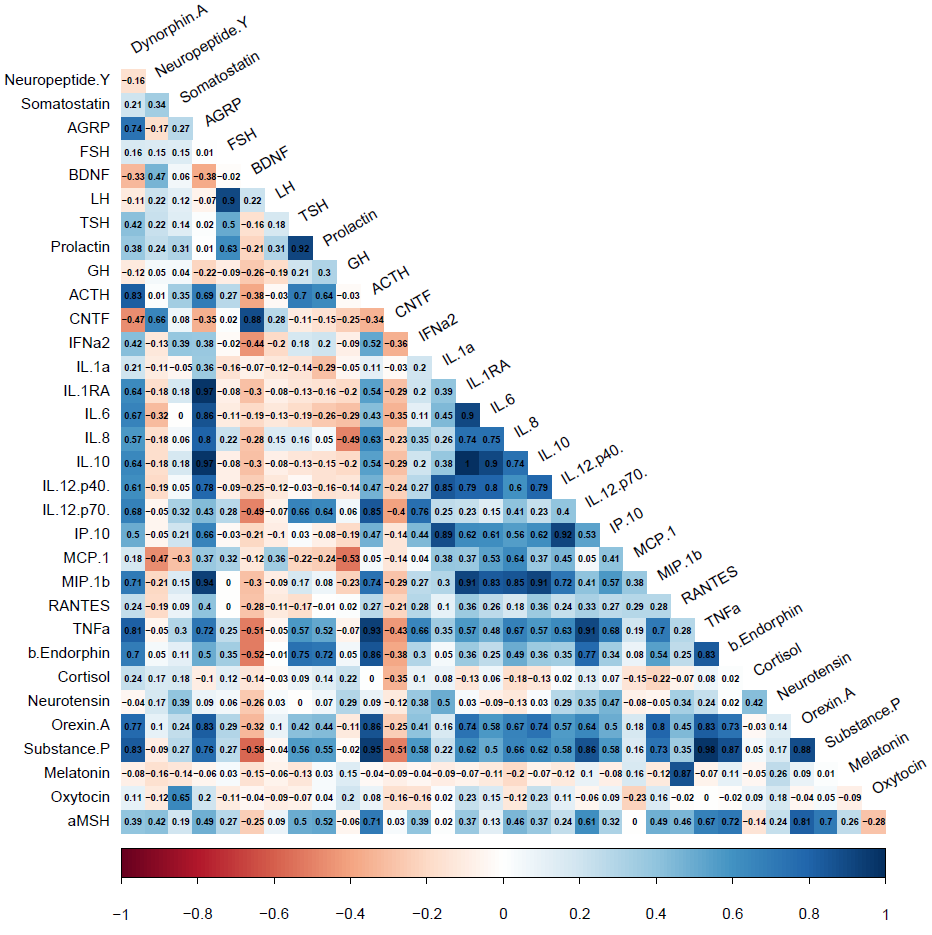


Figure 4. Visual representation of the direction and strength of the Pearson’s correlation coefficients between analytes assayed within the subgroup without seizures. Positive (blue), negative (red), strong (dark shading), and weak (light shading) correlations are depicted.

Table 8A: Pearson’s correlation coefficients between pairs of analytes within the subgroup without seizures. Complete dataset of significant correlations (p ≤ 0.05) after controlling for false discovery rate are presented.

| **Analyte 1** | **Analyte 2** | **Correlation** | **CI** | **Adj P** |
| --- | --- | --- | --- | --- |
| Dynorphin A | AGRP | 0.74 | [0.38, 0.90] | 0.011 |
| Dynorphin A | ACTH | 0.83 | [0.56, 0.94] | 0.001 |
| Dynorphin A | IL-1ra | 0.64 | [0.21, 0.86] | 0.048 |
| Dynorphin A | IL-6 | 0.67 | [0.25, 0.87] | 0.036 |
| Dynorphin A | IL-10 | 0.64 | [0.21, 0.86] | 0.048 |
| Dynorphin A | IL-12.p70 | 0.68 | [0.27, 0.88] | 0.032 |
| Dynorphin A | MIP-1β | 0.71 | [0.33, 0.89] | 0.019 |
| Dynorphin A | TNFα | 0.81 | [0.53, 0.93] | 0.002 |
| Dynorphin A | β endorphin | 0.70 | [0.31, 0.89] | 0.022 |
| Dynorphin A | Orexin A | 0.77 | [0.45, 0.92] | 0.005 |
| Dynorphin A | substance P | 0.83 | [0.57, 0.94] | 0.001 |
| Neuropeptide Y | CNTF | 0.66 | [0.24, 0.87] | 0.040 |
| Somatostatin | Oxytocin | 0.65 | [0.22, 0.87] | 0.046 |
| AGRP | ACTH | 0.69 | [0.30, 0.88] | 0.024 |
| AGRP | IL-1ra | 0.97 | [0.92, 0.99] | < .001 |
| AGRP | IL-6 | 0.86 | [0.64, 0.95] | < .001 |
| AGRP | IL-8 | 0.80 | [0.51, 0.93] | 0.003 |
| AGRP | IL-10 | 0.97 | [0.92, 0.99] | < .001 |
| AGRP | IL-12.p40 | 0.78 | [0.47, 0.92] | 0.004 |
| AGRP | IP-10 | 0.66 | [0.25, 0.87] | 0.038 |
| AGRP | MIP-1β | 0.94 | [0.83, 0.98] | < .001 |
| AGRP | TNFα | 0.72 | [0.36, 0.90] | 0.014 |
| AGRP | Orexin A | 0.83 | [0.57, 0.94] | 0.001 |
| AGRP | substance P | 0.76 | [0.43, 0.91] | 0.007 |
| FSH | LH | 0.90 | [0.73, 0.97] | < .001 |
| BDNF | CNTF | 0.88 | [0.68, 0.96] | < .001 |
| TSH | Prolactin | 0.92 | [0.77, 0.97] | < .001 |
| TSH | ACTH | 0.70 | [0.31, 0.89] | 0.022 |
| TSH | IL-12.p70 | 0.66 | [0.25, 0.87] | 0.038 |
| TSH | β endorphin | 0.75 | [0.40, 0.91] | 0.010 |
| Prolactin | ACTH | 0.64 | [0.21, 0.86] | 0.048 |
| Prolactin | IL-12.p70 | 0.64 | [0.21, 0.86] | 0.048 |
| Prolactin | β endorphin | 0.72 | [0.35, 0.90] | 0.015 |
| ACTH | IL-12.p70 | 0.85 | [0.60, 0.95] | 0.001 |
| ACTH | MIP-1β | 0.74 | [0.39, 0.91] | 0.011 |
| ACTH | TNFα | 0.93 | [0.81, 0.98] | < .001 |
| ACTH | β endorphin | 0.86 | [0.63, 0.95] | < .001 |
| ACTH | Orexin A | 0.86 | [0.65, 0.95] | < .001 |
| ACTH | substance P | 0.95 | [0.85, 0.98] | < .001 |
| ACTH | αMSH | 0.71 | [0.33, 0.89] | 0.018 |
| IFNα2 | IL-12.p70 | 0.76 | [0.42, 0.91] | 0.008 |
| IFNα2 | TNFα | 0.66 | [0.24, 0.87] | 0.040 |
| IL-1α | IL-12.p40 | 0.85 | [0.60, 0.94] | 0.001 |
| IL-1α | IP-10 | 0.89 | [0.70, 0.96] | < .001 |
| IL-1ra | IL-6 | 0.90 | [0.74, 0.97] | < .001 |
| IL-1ra | IL-8 | 0.74 | [0.39, 0.90] | 0.011 |
| IL-1ra | IL-10 | 1.00 | [1.00, 1.00] | < .001 |
| IL-1ra | IL-12.p40 | 0.79 | [0.49, 0.93] | 0.003 |
| IL-1ra | MIP-1β | 0.91 | [0.75, 0.97] | < .001 |
| IL-1ra | Orexin A | 0.74 | [0.38, 0.90] | 0.011 |
| IL-6 | IL-8 | 0.75 | [0.40, 0.91] | 0.010 |
| IL-6 | IL-10 | 0.90 | [0.74, 0.97] | < .001 |
| IL-6 | IL-12.p40 | 0.80 | [0.49, 0.93] | 0.003 |
| IL-6 | MIP-1β | 0.83 | [0.58, 0.94] | 0.001 |
| IL-8 | IL-10 | 0.74 | [0.39, 0.90] | 0.011 |
| IL-8 | MCP-1 | 0.64 | [0.21, 0.86] | 0.048 |
| IL-8 | MIP-1β | 0.85 | [0.62, 0.95] | 0.001 |
| IL-8 | TNFα | 0.67 | [0.27, 0.88] | 0.032 |
| IL-8 | Orexin A | 0.67 | [0.25, 0.87] | 0.036 |
| IL-8 | substance P | 0.66 | [0.24, 0.87] | 0.040 |
| IL-10 | IL-12.p40 | 0.79 | [0.48, 0.92] | 0.004 |
| IL-10 | MIP-1β | 0.91 | [0.76, 0.97] | < .001 |
| IL-10 | Orexin A | 0.74 | [0.38, 0.90] | 0.011 |
| IL-12.p40 | IP-10 | 0.92 | [0.78, 0.97] | < .001 |
| IL-12.p40 | MIP-1β | 0.72 | [0.35, 0.90] | 0.015 |
| IL-12.p70 | TNFα | 0.91 | [0.75, 0.97] | < .001 |
| IL-12.p70 | β endorphin | 0.77 | [0.45, 0.92] | 0.005 |
| IL-12.p70 | Orexin A | 0.64 | [0.21, 0.86] | 0.048 |
| IL-12.p70 | substance P | 0.86 | [0.64, 0.95] | < .001 |
| IP-10 | TNFα | 0.68 | [0.28, 0.88] | 0.028 |
| MIP-1β | TNFα | 0.70 | [0.31, 0.89] | 0.021 |
| MIP-1β | Orexin A | 0.80 | [0.50, 0.93] | 0.003 |
| MIP-1β | substance P | 0.73 | [0.38, 0.90] | 0.011 |
| RANTES | Melatonin | 0.87 | [0.65, 0.95] | < .001 |
| TNFα | β endorphin | 0.83 | [0.57, 0.94] | 0.001 |
| TNFα | Orexin A | 0.83 | [0.56, 0.94] | 0.001 |
| TNFα | substance P | 0.98 | [0.93, 0.99] | < .001 |
| TNFα | αMSH | 0.67 | [0.26, 0.88] | 0.034 |
| β endorphin | Orexin A | 0.73 | [0.38, 0.90] | 0.011 |
| β endorphin | substance P | 0.87 | [0.66, 0.95] | < .001 |
| β endorphin | αMSH | 0.72 | [0.34, 0.89] | 0.016 |
| Orexin A | substance P | 0.88 | [0.69, 0.96] | < .001 |
| Orexin A | αMSH | 0.81 | [0.54, 0.93] | 0.002 |
| substance P | αMSH | 0.70 | [0.32, 0.89] | 0.021 |

Table 8B. Significant Pearson’s correlations (p ≤ 0.001) between analyte pairs in the subgroup without seizures. There were 30 unique positive analyte correlations (bold font) specific to those without seizures that were not significant within the subgroup with seizures.

| **Analyte 1** | **Analyte 2** | **Correlation** | **CI** | **Adj P** |
| --- | --- | --- | --- | --- |
| **Dynorphin A** | **ACTH** | **0.83** | **[0.56, 0.94]** | **0.001** |
| **Dynorphin A** | **substance P** | **0.83** | **[0.57, 0.94]** | **0.001** |
| **AGRP** | **Orexin A** | **0.83** | **[0.57, 0.94]** | **0.001** |
| **ACTH** | **IL-12.p70** | **0.85** | **[0.60, 0.95]** | **0.001** |
| **IL-1α** | **IL-12.p40** | **0.85** | **[0.60, 0.94]** | **0.001** |
| **IL-6** | **MIP-1β** | **0.83** | **[0.58, 0.94]** | **0.001** |
| **IL-8** | **MIP-1β** | **0.85** | **[0.62, 0.95]** | **0.001** |
| **TNFα** | **β endorphin** | **0.83** | **[0.57, 0.94]** | **0.001** |
| **TNFα** | **Orexin A** | **0.83** | **[0.56, 0.94]** | **0.001** |
| **AGRP** | **IL-1ra** | **0.97** | **[0.92, 0.99]** | **< .001** |
| **AGRP** | **IL-6** | **0.86** | **[0.64, 0.95]** | **< .001** |
| **AGRP** | **IL-10** | **0.97** | **[0.92, 0.99]** | **< .001** |
| **AGRP** | **MIP-1β** | **0.94** | **[0.83, 0.98]** | **< .001** |
| **FSH** | **LH** | **0.90** | **[0.73, 0.97]** | **< .001** |
| **BDNF** | **CNTF** | **0.88** | **[0.68, 0.96]** | **< .001** |
| **TSH** | **Prolactin** | **0.92** | **[0.77, 0.97]** | **< .001** |
| **ACTH** | **TNFα** | **0.93** | **[0.81, 0.98]** | **< .001** |
| **ACTH** | **β endorphin** | **0.86** | **[0.63, 0.95]** | **< .001** |
| **ACTH** | **Orexin A** | **0.86** | **[0.65, 0.95]** | **< .001** |
| **ACTH** | **substance P** | **0.95** | **[0.85, 0.98]** | **< .001** |
| **IL-1α** | **IP-10** | **0.89** | **[0.70, 0.96]** | **< .001** |
| **IL-1ra** | **IL-6** | **0.90** | **[0.74, 0.97]** | **< .001** |
| IL-1ra | IL-10 | 1.00 | [1.00, 1.00] | < .001 |
| IL-1ra | MIP-1β | 0.91 | [0.75, 0.97] | < .001 |
| **IL-6** | **IL-10** | **0.90** | **[0.74, 0.97]** | **< .001** |
| IL-10 | MIP-1β | 0.91 | [0.76, 0.97] | < .001 |
| **IL-12.p40** | **IP-10** | **0.92** | **[0.78, 0.97]** | **< .001** |
| **IL-12.p70** | **TNFα** | **0.91** | **[0.75, 0.97]** | **< .001** |
| **IL-12.p70** | **substance P** | **0.86** | **[0.64, 0.95]** | **< .001** |
| **RANTES** | **Melatonin** | **0.87** | **[0.65, 0.95]** | **< .001** |
| **TNFα** | **substance P** | **0.98** | **[0.93, 0.99]** | **< .001** |
| **β endorphin** | **substance P** | **0.87** | **[0.66, 0.95]** | **< .001** |
| **Orexin A** | **substance P** | **0.88** | **[0.69, 0.96]** | **< .001** |


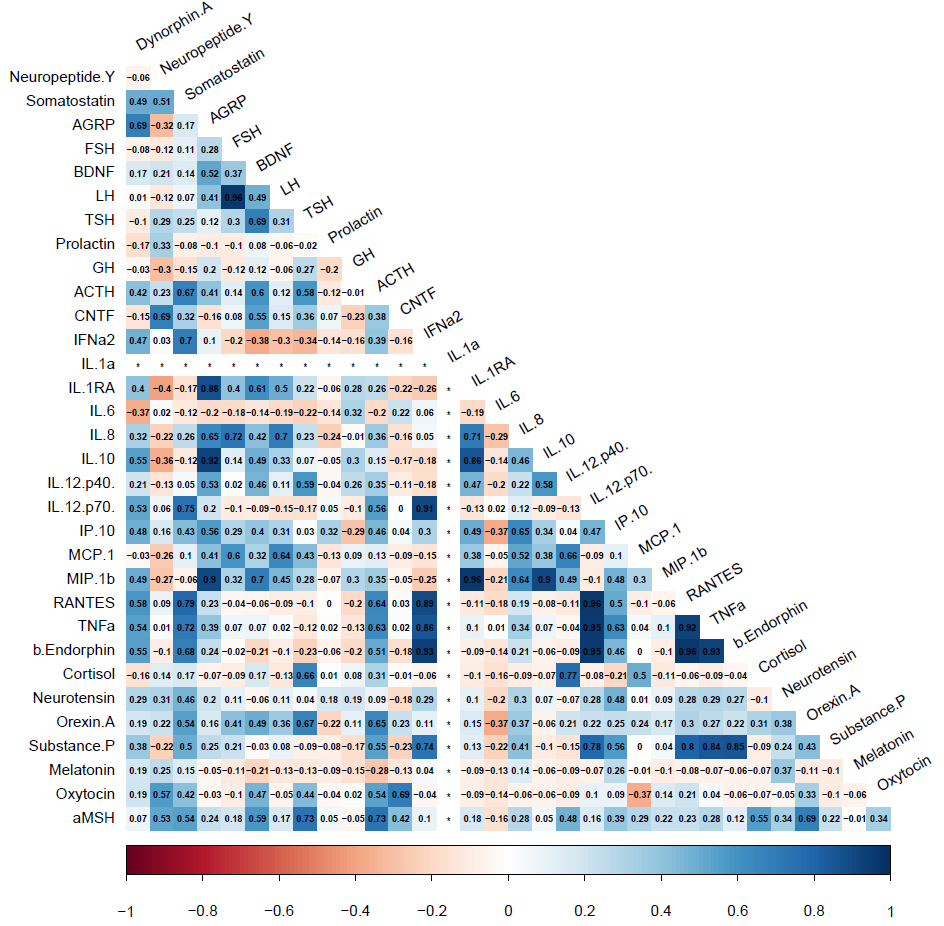


Figure 5. Visual representation of the direction and strength of the Pearson’s correlation coefficients between analytes assayed within the subgroup with quadriplegia. Positive (blue), negative (red), strong (dark shading), and weak (light shading) correlations are depicted.

Table 9A: Pearson’s correlation coefficients between pairs of analytes within the subgroup with quadriplegia. Complete dataset of significant correlations (p ≤ 0.05) after controlling for false discovery rate are presented.

| **Analyte 1** | **Analyte 2** | **Correlation** | **CI** | **Adj P** |
| --- | --- | --- | --- | --- |
| Dynorphin A | AGRP | 0.69 | [0.31, 0.88] | 0.030 |
| Neuropeptide Y | CNTF | 0.69 | [0.31, 0.88] | 0.030 |
| Somatostatin | ACTH | 0.67 | [0.28, 0.87] | 0.039 |
| Somatostatin | IFNα2 | 0.70 | [0.33, 0.88] | 0.028 |
| Somatostatin | IL-12.p70 | 0.75 | [0.42, 0.90] | 0.011 |
| Somatostatin | RANTES | 0.79 | [0.50, 0.92] | 0.004 |
| Somatostatin | TNFα | 0.72 | [0.36, 0.89] | 0.020 |
| Somatostatin | β endorphin | 0.68 | [0.30, 0.88] | 0.032 |
| AGRP | IL-1ra | 0.88 | [0.68, 0.96] | < .001 |
| AGRP | IL-10 | 0.92 | [0.79, 0.97] | < .001 |
| AGRP | MIP-1β | 0.90 | [0.74, 0.96] | < .001 |
| FSH | LH | 0.96 | [0.90, 0.99] | < .001 |
| FSH | IL-8 | 0.72 | [0.37, 0.89] | 0.018 |
| BDNF | TSH | 0.69 | [0.3, 0.88] | 0.031 |
| BDNF | MIP-1β | 0.70 | [0.33, 0.88] | 0.028 |
| LH | IL-8 | 0.70 | [0.33, 0.88] | 0.028 |
| TSH | Cortisol | 0.66 | [0.26, 0.87] | 0.045 |
| TSH | Orexin A | 0.67 | [0.28, 0.87] | 0.041 |
| TSH | αMSH | 0.73 | [0.39, 0.90] | 0.016 |
| ACTH | αMSH | 0.73 | [0.39, 0.90] | 0.016 |
| CNTF | Oxytocin | 0.69 | [0.31, 0.88] | 0.030 |
| IFNα2 | IL-12.p70 | 0.91 | [0.76, 0.97] | < .001 |
| IFNα2 | RANTES | 0.89 | [0.72, 0.96] | < .001 |
| IFNα2 | TNFα | 0.86 | [0.64, 0.95] | < .001 |
| IFNα2 | β endorphin | 0.93 | [0.82, 0.98] | < .001 |
| IFNα2 | substance P | 0.74 | [0.40, 0.90] | 0.014 |
| IL-1ra | IL-8 | 0.71 | [0.34, 0.89] | 0.024 |
| IL-1ra | IL-10 | 0.86 | [0.64, 0.95] | < .001 |
| IL-1ra | MIP-1β | 0.96 | [0.90, 0.99] | < .001 |
| IL-10 | MIP-1β | 0.90 | [0.74, 0.96] | < .001 |
| IL-12.p40 | MCP-1 | 0.66 | [0.26, 0.87] | 0.045 |
| IL-12.p40 | Cortisol | 0.77 | [0.46, 0.91] | 0.007 |
| IL-12.p70 | RANTES | 0.96 | [0.90, 0.99] | < .001 |
| IL-12.p70 | TNFα | 0.95 | [0.87, 0.98] | < .001 |
| IL-12.p70 | β endorphin | 0.95 | [0.86, 0.98] | < .001 |
| IL-12.p70 | substance P | 0.78 | [0.48, 0.92] | 0.005 |
| RANTES | TNFα | 0.92 | [0.80, 0.97] | < .001 |
| RANTES | β endorphin | 0.96 | [0.89, 0.99] | < .001 |
| RANTES | substance P | 0.80 | [0.52, 0.93] | 0.003 |
| TNFα | β endorphin | 0.93 | [0.80, 0.97] | < .001 |
| TNFα | substance P | 0.84 | [0.61, 0.94] | 0.001 |
| β endorphin | substance P | 0.85 | [0.63, 0.95] | < .001 |
| Orexin A | αMSH | 0.69 | [0.31, 0.88] | 0.030 |

Table 9B. Significant Pearson’s correlations (p ≤ 0.001) between analyte pairs in the subgroup with quadriplegia. There were 16 unique positive analyte correlations (bold font) specific to those with quadriplegia that were not significant within the subgroup without quadriplegia.

| **Analyte 1** | **Analyte 2** | **Correlation** | **CI** | **Adj P** |
| --- | --- | --- | --- | --- |
| **TNFα** | **substance P** | **0.84** | **[0.61, 0.94]** | **0.001** |
| **AGRP** | **IL-1ra** | **0.88** | **[0.68, 0.96]** | **< .001** |
| AGRP | IL-10 | 0.92 | [0.79, 0.97] | < .001 |
| AGRP | MIP-1β | 0.9 | [0.74, 0.96] | < .001 |
| FSH | LH | 0.96 | [0.90, 0.99] | < .001 |
| **IFNα2** | **IL-12.p70** | **0.91** | **[0.76, 0.97]** | **< .001** |
| **IFNα2** | **RANTES** | **0.89** | **[0.72, 0.96]** | **< .001** |
| **IFNα2** | **TNFα** | **0.86** | **[0.64, 0.95]** | **< .001** |
| **IFNα2** | **β endorphin** | **0.93** | **[0.82, 0.98]** | **< .00** |
| **IL-1ra** | **IL-10** | **0.86** | **[0.64, 0.95]** | **< .001** |
| **IL-1ra** | **MIP-1β** | **0.96** | **[0.90, 0.99]** | **< .001** |
| **IL-10** | **MIP-1β** | **0.90** | **[0.74, 0.96]** | **< .001** |
| **IL-12.p70** | **RANTES** | **0.96** | **[0.90, 0.99]** | **< .001** |
| **IL-12.p70** | **TNFα** | **0.95** | **[0.87, 0.98]** | **< .001** |
| **IL-12.p70** | **β endorphin** | **0.95** | **[0.86, 0.98]** | **< .001** |
| **RANTES** | **TNFα** | **0.92** | **[0.80, 0.97]** | **< .001** |
| **RANTES** | **β endorphin** | **0.96** | **[0.89, 0.99]** | **< .001** |
| **TNFα** | **β endorphin** | **0.93** | **[0.80, 0.97]** | **< .001** |
| **β endorphin** | **substance P** | **0.85** | **[0.63, 0.95]** | **< .001** |


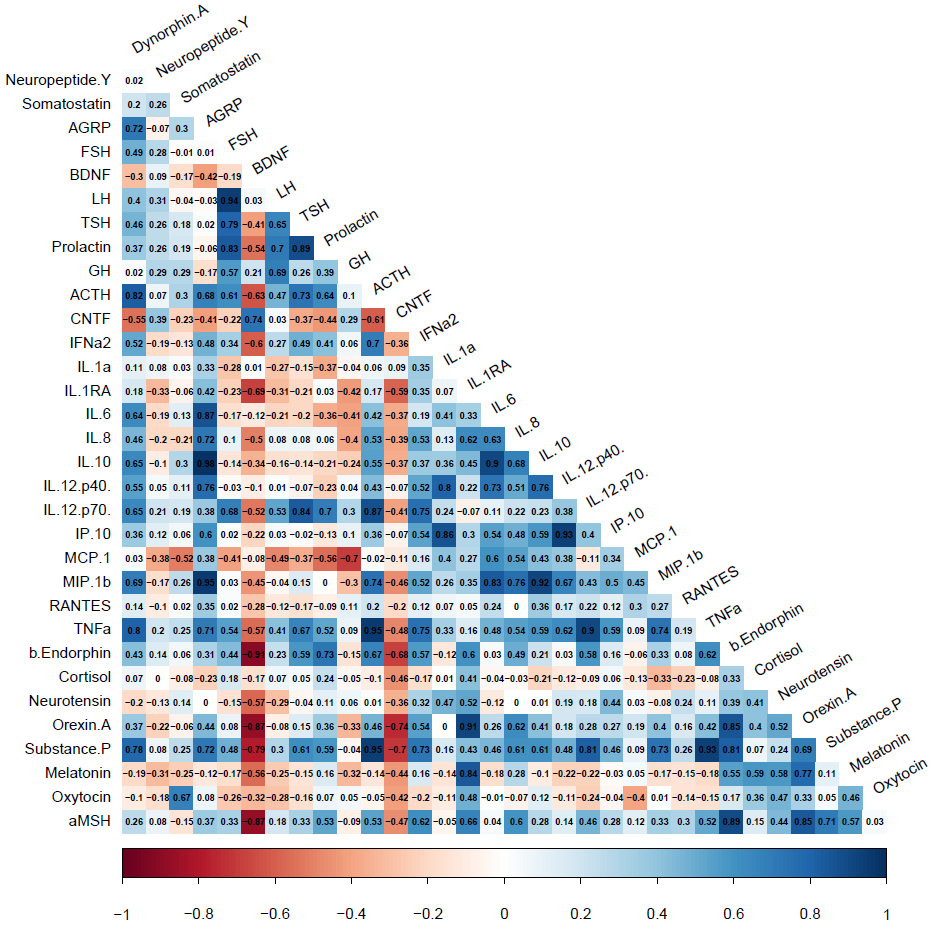


Figure 6. Visual representation of the direction and strength of the Pearson’s correlation coefficients between analytes assayed within the subgroup without quadriplegia. Positive (blue), negative (red), strong (dark shading), and weak (light shading) correlations are depicted.

Table 10A: Pearson’s correlation coefficients between pairs of analytes within the subgroup without quadriplegia. Complete dataset of significant correlations (p ≤ 0.05) after controlling for false discovery rate are presented.

| **Analyte 1** | **Analyte 2** | **Correlation** | **CI** | **Adj P** |
| --- | --- | --- | --- | --- |
| Dynorphin A | ACTH | 0.82 | [0.44, 0.95] | 0.037 |
| AGRP | IL-6 | 0.87 | [0.55, 0.96] | 0.017 |
| AGRP | IL-10 | 0.98 | [0.94, 1.00] | < .001 |
| AGRP | MIP-1β | 0.95 | [0.82, 0.99] | 0.001 |
| FSH | LH | 0.94 | [0.79, 0.99] | 0.001 |
| FSH | Prolactin | 0.83 | [0.47, 0.96] | 0.030 |
| BDNF | β endorphin | -0.91 | [-0.98, -0.68] | 0.006 |
| BDNF | Orexin A | -0.87 | [-0.97, -0.58] | 0.014 |
| BDNF | αMSH | -0.87 | [-0.97, -0.57] | 0.014 |
| TSH | Prolactin | 0.89 | [0.62, 0.97] | 0.010 |
| TSH | IL-12.p70 | 0.84 | [0.47, 0.96] | 0.030 |
| ACTH | IL-12.p70 | 0.87 | [0.57, 0.97] | 0.014 |
| ACTH | TNFα | 0.95 | [0.82, 0.99] | 0.001 |
| ACTH | substance P | 0.95 | [0.80, 0.99] | 0.001 |
| IL-1α | IP-10 | 0.86 | [0.55, 0.96] | 0.017 |
| IL-1ra | Orexin A | 0.91 | [0.70, 0.98] | 0.005 |
| IL-1ra | Melatonin | 0.84 | [0.49, 0.96] | 0.027 |
| IL-6 | IL-10 | 0.90 | [0.66, 0.97] | 0.007 |
| IL-6 | MIP-1β | 0.83 | [0.47, 0.96] | 0.030 |
| IL-10 | MIP-1β | 0.92 | [0.70, 0.98] | 0.005 |
| IL-12.p40 | IP-10 | 0.93 | [0.76, 0.98] | 0.002 |
| IL-12.p70 | TNFα | 0.90 | [0.65, 0.97] | 0.007 |
| IL-12.p70 | substance P | 0.81 | [0.42, 0.95] | 0.044 |
| TNFα | substance P | 0.93 | [0.76, 0.98] | 0.002 |
| β endorphin | Orexin A | 0.85 | [0.51, 0.96] | 0.024 |
| β endorphin | substance P | 0.81 | [0.41, 0.95] | 0.045 |
| β endorphin | αMSH | 0.89 | [0.62, 0.97] | 0.010 |
| Orexin A | αMSH | 0.85 | [0.51, 0.96] | 0.024 |

Table 10B. Significant Pearson’s correlations (p ≤ 0.001) between analyte pairs in the subgroup without quadriplegia. There were 2 unique positive analyte correlations (bold font) specific to those without quadriplegia that were not significant within the subgroup with quadriplegia.

| **Analyte 1** | **Analyte 2** | **Correlation** | **CI** | **Adj. p** |
| --- | --- | --- | --- | --- |
| AGRP | MIP-1β | 0.95 | [0.82, 0.99] | 0.001 |
| FSH | LH | 0.94 | [0.79, 0.99] | 0.001 |
| **ACTH** | **TNFα** | **0.95** | **[0.82, 0.99]** | **0.001** |
| **ACTH** | **substance P** | **0.95** | **[0.80, 0.99]** | **0.001** |
| AGRP | IL-10 | 0.98 | [0.94, 1.00] | < .001 |
